# Artificial intelligence in cardiovascular care: a systematic review and meta-analysis of randomised controlled trials

**DOI:** 10.1016/j.eclinm.2026.104123

**Published:** 2026-08-06

**Authors:** Angus Qi Chwen Ong, Chin-Siang Ang, Iva Bojic, Casey L. Johnson, Hesham Aggour, Fu Siong Ng, Josip Car, Paul Leeson, Judite Gonçalves, Nai Ming Lai

**Affiliations:** a School of Public Health, Imperial College London, London, UK; b Oxford Cardiovascular Clinical Research Facility, Division of Cardiovascular Medicine, Radcliffe Department of Medicine, University of Oxford, Oxford, UK; c Lee Kong Chian School of Medicine, Nanyang Technological University, Singapore, Singapore; d National Heart and Lung Institute, Imperial College London, London, UK; e King's Population Health Institute, King's College London, London, UK; f School of Life Course & Population Sciences, King's College London, London, UK; g School of Medicine, Faculty of Health and Medical Sciences, Taylor's University, Subang Jaya, Malaysia

**Keywords:** Artificial intelligence, Machine learning, Cardiovascular disease, Cardiology, Clinical trial, Evidence synthesis

## Abstract

**Background:**

Artificial intelligence (AI) holds potential to transform cardiovascular care, but evidence on its effectiveness in clinical practice remains inconsistent. We aimed to synthesise evidence from randomised controlled trials (RCTs) on the effectiveness of AI-enabled cardiovascular care, summarise the trial design and characteristics of AI systems, and evaluate methodological quality and reporting transparency.

**Methods:**

In this systematic review and meta-analysis, we searched Embase, MEDLINE, Scopus, Cochrane Central Register of Controlled Trials, and ClinicalTrials.gov for RCTs that evaluated effectiveness of AI interventions in cardiovascular care, published in English from database inception to July 07, 2025. The search was updated on April 28, 2026. We followed Cochrane guidance for study selection and data extraction. Risk of bias was assessed using Cochrane's Risk of Bias tools and reporting transparency using CONSORT-AI checklist. We calculated summary effects using inverse-variance-weighted random-effects meta-analyses and assessed the certainty of evidence using GRADE. Between-study heterogeneity was quantified using χ^2^ (Cochran's Q) test and I^2^ statistic. Publication bias was not assessed due to small number of studies. This study was registered with PROSPERO (CRD420251090250).

**Findings:**

Of 12,217 records identified, 31 RCTs from 13 regions (n = 1,685,717 patients) were included in the systematic review, and 11 of these (n = 1,614,689) in the meta-analysis. Most RCTs were published after 2021 (90%), multicentre (58%), and had short follow-up duration (<12 months; 65%). Risk of bias was low in seven trials (23%), and overall reporting transparency was moderate. Twenty-two trials (71%) reported significant benefit of AI interventions on primary endpoints, mostly intermediate process measures, while nine trials (29%) found no significant effect. Compared with routine care, image-based AI-clinical decision support system had significant effect on major adverse cardiovascular events (risk ratio [RR] 0.74 [95% CI 0.58–0.96]; I^2^ = 0%). AI-based mobile health interventions showed reduction in systolic blood pressure (mean difference −3.18 mm Hg [95% CI −5.50 to −0.86]; I^2^ = 0%), but not in diastolic blood pressure and body mass index. AI-enhanced electrocardiograms did not have significant effect on heart failure detection (RR 1.22 [0.70–2.14]; I^2^ = 90%). AI had unclear effects on atrial fibrillation diagnosis and echocardiogram utilisation due to very low certainty evidence. Certainty of evidence ranged from low to very low across outcomes due to risk of bias, indirectness, and imprecision.

**Interpretation:**

AI demonstrated potential benefits in intermediate process measures in cardiovascular care but evidence of its effect on hard clinical outcomes remains limited. Current evidence is constrained by high risk of bias, trial heterogeneity, inadequate reporting of AI-specific components, limited generalisability, and a scarcity of trials with clinically meaningful endpoints. More rigorous, representative, and transparently reported RCTs with longer follow-up and long-term clinical outcomes are needed.

**Funding:**

None received.

**Research in context**

**Evidence before this study**

We searched Ovid Embase, Ovid MEDLINE, Scopus, and the Cochrane Central Register of Controlled Trials (CENTRAL) from database inception to 07 July, 2025, using MeSH terms and a combination of keywords related to artificial intelligence (AI) and cardiovascular disease. No restrictions were applied on language or publication type. We identified multiple clinical trials evaluating AI interventions in patients with or at risk of cardiovascular disease, as well as AI interventions supporting healthcare professionals involved in cardiovascular care. Previous evidence syntheses in this area include a scoping review of prospective human validation studies of AI models in cardiology, which searched two databases only and combined heterogeneous study designs. Other systematic and scoping reviews have focused on randomised controlled trials but adopted a broad scope across a range of clinical specialities and patient populations, limiting their ability to quantitatively synthesise findings within cardiovascular care. To our knowledge, no systematic review and meta-analysis has comprehensively synthesised evidence exclusively from randomised controlled trials to determine the effectiveness of AI interventions in cardiovascular care compared with usual care, alongside an assessment of trial methodological quality and reporting practices.

**Added value of this study**

To our knowledge, this is the first systematic review and meta-analysis to assess the effectiveness of AI interventions in cardiovascular care using evidence from randomised controlled trials. We synthesised results from 31 trials published between 2017 and 2026, encompassing 1,685,717 participants across a range of cardiovascular conditions, including atrial fibrillation, coronary artery disease, hypertension, and heart failure. Most trials compared AI interventions with usual care. Over two-thirds reported significant effects of AI on their primary endpoints, which were mostly intermediate process measures. Meta-analysis of appropriate data showed that, compared with usual care, AI interventions were associated with statistically significant differences in major adverse cardiovascular events and systolic blood pressure. No significant difference was observed in diastolic blood pressure, body mass index, and heart failure diagnosis. Evidence for effects on atrial fibrillation diagnosis and echocardiogram utilisation was uncertain. Across outcomes, the certainty of evidence ranged from low to very low, primarily due to potential risk of bias, indirectness, and imprecision. Only a quarter of included trials were judged at low risk of bias, with common concerns related to outcome measurement and deviations from intended interventions. Trials evaluating AI in cardiovascular care had moderate adherence to AI-specific reporting items in CONSORT-AI.

**Implications of all the available evidence**

AI in cardiovascular care demonstrated potential benefits in improving intermediate process measures and some patient health outcomes, but overall evidence on hard clinical outcomes remain limited. Current evidence base is characterised by short follow-up durations, trial heterogeneity, considerable risk of bias, low certainty of evidence, and limited use of patient-centred clinical endpoints. Additional, rigorous, inclusive, and transparently reported RCTs with longer follow-up are needed to determine whether improvements in surrogate endpoints or care processes translate into meaningful and sustained clinical benefit.

## Introduction

Artificial intelligence (AI) holds significant potential to transform cardiovascular care, particularly when applied to widely used tools such as electrocardiogram (ECG) and echocardiography.^1^^,^^2^ Its ability to harness complex and abundant data could improve detection, risk stratification, and personalised management of cardiovascular disease (CVD) and its risk factors.^3^, ^4^, ^5^, ^6^ Studies that directly compared AI with human performance have been promising and widely used to justify implementation of AI-enabled technologies.^7^ However, these studies were mostly retrospective or proof-of-concept evaluations,^8^^,^^9^ using surrogate endpoint outcomes with limited comparability to real-world clinical practice.^10^ As a result there is growing recognition of the need for prospective, randomised evaluation of AI technologies to generate meaningful and robust evidence on how AI impacts patient outcome.^11^

Registered clinical trials of AI-enabled medical devices are increasing,^12^ and in response several systematic reviews have synthesised evidence from randomised controlled trials (RCTs).^13^, ^14^, ^15^ These reviews reported positive effects of AI interventions on study endpoints in other clinical domains, notably within gastroenterology, anaesthesiology and radiology.^13^^,^^15^, ^16^, ^17^ Given the high global burden of CVD and rapid expansion of AI technologies in cardiology, similar evidence synthesis efforts are needed to provide clinicians, patients, policymakers, and other stakeholders with a consolidated understanding of where AI delivers benefit in cardiovascular care. While there have been numerous narrative reviews,^2^^,^^6^^,^^18^^,^^19^ only a limited number of systematic reviews are available. One characterised prospective human validation studies of AI models in cardiology but was limited to two databases and combined heterogeneous study designs.^20^

Therefore we undertook a systematic review and meta-analysis to establish a robust evidence base to inform clinical practice, guide the design of future trials, and support the responsible integration of AI into cardiovascular care. We restricted our review to RCTs and employed a comprehensive search strategy to maximise study retrieval. To ensure a comprehensive appraisal of effectiveness and gain insight into methodological rigour and reporting transparency, we evaluated trial quality and adherence to the Consolidated Standards of Reporting Trials-Artificial Intelligence (CONSORT-AI) reporting guidelines.^21^

## Methods

This systematic review was conducted in accordance with the recommendations from Cochrane Handbook for Systematic Reviews of Interventions and reported following the Preferred Reporting Items for Systematic Reviews and Meta-analyses (PRISMA) reporting guidelines (Tables S1 and S2).^22^^,^^23^ The systematic review protocol was registered on PROSPERO (CRD420251090250).^24^

### Search strategy and selection criteria

We developed a search strategy in consultation with a senior medical librarian. Searches were performed on Ovid Embase, Ovid MEDLINE, Scopus, Cochrane Central Register of Controlled Trials (CENTRAL), and ClinicalTrials.gov for articles published in English from database inception to July 07, 2025. This search was updated on April 27, 2026. We used both Medical Subject Heading terms and keywords related to artificial intelligence, cardiovascular disease, cardiology, and randomised controlled trials. The complete search strategy is in Expanded Method S1. We also performed supplementary searches for up to first 10 pages of Google Scholar using combinations of pertinent keywords. We further conducted forward and backward citation searches of included studies using citationchaser.^25^ Both Google Scholar and citation searches were first completed on August 21, 2025, and updated on May 08, 2026.

Search results from all databases were first downloaded and imported into Endnote 21 to form a single combined library. Citations were subsequently uploaded to Covidence (Veritas Health Innovation, Melbourne, Australia) for automated deduplication. We identified eligible articles through a two-stage process: (1) Title and abstract screening, (2) Full-text review. Titles and abstracts were screened against pre-defined eligibility criteria (Expanded Method S2). One author (AQCO) screened all records, while three other authors (CSA, IB, and NML) each screened a subset of the records such that each record was reviewed independently by at least two authors. Subsequently, full texts of retained records were retrieved and independently reviewed by at least two authors. Discrepancies were resolved through discussion or by involving a third author to establish consensus. Reasons for exclusion at the full-text review stage were documented.

### Data extraction

A pre-specified data extraction form was developed on Microsoft Excel in accordance with the study objectives and the guidelines from the Cochrane Handbook for Systematic Reviews of Interventions.^22^ The form was piloted on approximately 20% of included articles for two purposes: (i) ensure consistency and reproducibility; (ii) minimise discrepancies among reviewers. Following this, the form was refined and updated. Similar to article selection stage, data from each included article were extracted independently by at least two of four authors (AQCO, CSA, IB, and NML). Extracted data were compared to identify discrepancies, which were resolved through discussion or by involving a third author. Information extracted from studies included (i) publication metadata, (ii) study design (iii) study setting, (iv) participants' characteristics, (v) intervention characteristics, (vi) comparison, (vii) study outcomes, and (viii) funding information. We did not contact the study authors to request additional or clarify uncertain information.

### Statistical analysis

We tabulated qualitative data (e.g., types and features of AI interventions) of included studies along with a descriptive summary. For quantitative data such as participant demographics and intervention duration, we calculated descriptive statistics and reported median values with interquartile ranges. We performed meta-analyses only when comparable data were available for a given outcome. Meta-analyses were conducted using ReviewManager version 9.15.0 and in accordance with Cochrane guidance.^26^ We calculated summary effect estimates and 95% confidence intervals (CIs) with inverse variance weighted random-effects meta-analyses to account for clinical heterogeneity in AI intervention types and population characteristics. Event counts were extracted directly from the trial reports or derived from reported percentages. The relative intervention effects were described by risk ratios (RR) with 95% CIs and displayed in forest plots.

We quantified between-study heterogeneity using the χ^2^ (Cochran's Q) test and the I^2^ statistic. Statistical heterogeneity was considered substantial when I^2^ exceeded 50% and the p value for the χ^2^ test was less than 0.10. A higher p-value threshold than the conventional 0.05 was chosen because few trials were expected to contribute comparable outcomes, reducing the power of the heterogeneity test. We did not assess publication bias with funnel plots and Egger's regression test because these methods are considered unreliable for meta-analyses of outcomes with a small number (<10) of studies.^26^^,^^27^ Subgroup analyses and meta-regression were not undertaken for the same reason. However, as a post-hoc analysis, funnel plots were generated for visualisation purposes.

We conducted a series of sensitivity analyses to assess the robustness of pooled estimates. These included leave-one-out analyses, in which each study was sequentially excluded to evaluate whether any single trial disproportionately influenced the direction of associations, and comparisons of the results obtained using fixed-effect and random-effects models. For outcomes based on two or three studies only, we compared results of random-effects models estimated using the Wald-type and Hartung–Knapp–Sidik–Jonkman methods, as recommended.^26^ Sensitivity analyses excluding studies with high risk of bias were conducted as post-hoc analyses.

### Quality assessment of included studies and overall evidence

We assessed the risk of bias using the revised Cochrane risk-of-bias version 2 tool for randomised trials.^28^ As this review included individually randomised and cluster randomised trials, the pertinent version of the tool was used. We considered the following aspects: randomisation method, treatment allocation concealment, blinding of outcome assessors, completeness of outcome data, evidence of selective outcome reporting, other sources of bias, and appropriateness of statistical tests. Included articles were ranked independently by two authors (AQCO and CSA) into three categories: low risk of bias, some concerns, or high risk of bias. Additionally, two authors (AQCO and CLJ) evaluated trial adherence to the 14 AI-specific items in the CONSORT-AI reporting guidelines.^21^ Discrepancies were resolved through discussion or involvement of a third author (NML).

The certainty of evidence by outcome of interest was assessed by two authors (AQCO and NML) independently using the Grading of Recommendations, Assessment, Development, and Evaluation (GRADE) criteria.^29^ Given that observational studies were excluded, all studies included in the meta-analysis started at high certainty (equivalent to 4 points). The certainty of evidence may then be downgraded based on five domains (risk of bias, inconsistency, indirectness, imprecision, and publication bias). We did not use the three upgrading criteria (large effect size, dose–response gradient, and residual confounding) as they are usually used for non-randomised studies and applying them to RCTs may inflate the certainty of evidence.^30^ The quality of evidence was classified as high certainty (≥4 points overall), moderate certainty (3 points), low certainty (2 points), or very-low certainty (≤1).

### Ethics

This is a systematic review and meta-analysis of previously published studies. Ethics approval and signing of informed consent form were not required.

### Role of the funding source

No funding was received for this study. AQCO, CSA and NML had full access to all the data in the study. All authors shared final responsibility for the decision to submit for publication.

## Results

**Fig. 1:**
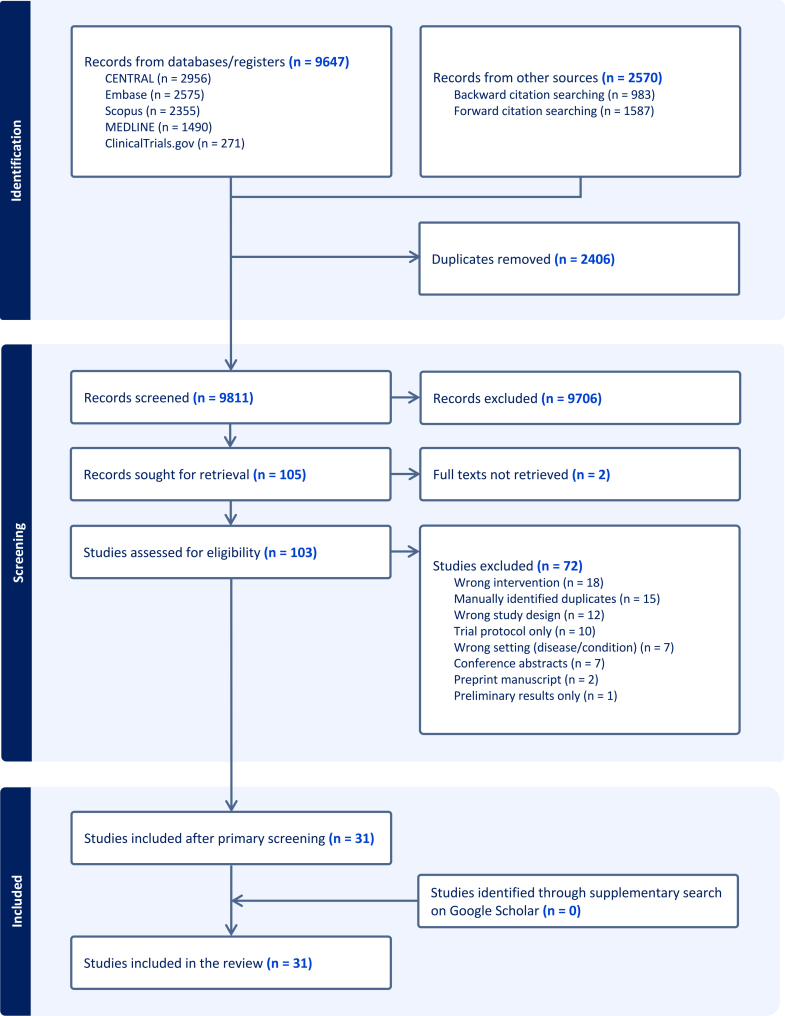
**Study selection process depicted in the PRISMA flow diagram.** PRISMA = Preferred Reporting Items for Systematic reviews and Meta-Analyses.

The electronic search retrieved 12,217 records, with 9811 remaining after removing duplicates. 9706 records were excluded after title and abstract screening, and 105 were retained for full-text review. Following this, 31 studies in total were included in this systematic review. Among these, 24 were identified from initial search.^31^, ^32^, ^33^, ^34^, ^35^, ^36^, ^37^, ^38^, ^39^, ^40^, ^41^, ^42^, ^43^, ^44^, ^45^, ^46^, ^47^, ^48^, ^49^, ^50^, ^51^, ^52^, ^53^, ^54^ The updated search, performed on April 28, 2026, yielded an additional seven studies that were eligible for inclusion.^55^, ^56^, ^57^, ^58^, ^59^, ^60^, ^61^ The reasons for exclusion were documented (Table S3 in Supplementary Material) and summarised (Fig. 1). We did not identify additional relevant study through supplementary search on Google Scholar or citation searching.

Characteristics of included RCTs are presented in Table 1. The included trials were published between 2017 and 2026, with the majority (28 [90%]) published after 2020. Only one relevant trial was identified in 2017,^52^ 2019,^54^ and 2020.^53^ Trials were predominantly conducted in multiple centres (18 [58%]) and in a single country (27 [87%]), mostly in the United States (11 trials [35%]), followed by China (Mainland; six trials), Taiwan (four trials), United Kingdom (three trials), Germany, Belgium, Japan, and France (two trials each), and Nigeria, South Korea, Australia, Denmark, and the Netherlands (one trial each). The only two multinational trials involved mainly European countries.^34^^,^^35^ Temporal and geographical distributions of included RCTs are shown in Figure S1 in Supplementary Material.

**Table 1:** Characteristics of included randomised controlled trials.

Study, year	Trial location	Study design; site; setting	Sample size	Sex (Female; Male, %)	Majority race (%)	Target condition	Intervention	Primary endpoint	Result	Effect size (95% CI)
Adedinsewo et al.,^31^ 2024	Nigeria	RCT; Multicentre; Hospital	1195	100.0; 0	Black (100.0)	Pregnancy-related left ventricular systolic dysfunction	AI-digital stethoscope and AI-ECG algorithm	Identification of LVSD	Significant improvement	AI-stethoscope: OR 2.12 (1.05–4.27); AI-ECG: OR 1.75 (0.85–3.62)
Andre et al.,^32^ 2023	Germany	RCT; Single centre; Hospital outpatient service	120	34.2; 65.8	NR	Coronary artery disease	AI CT angiography assessment	Coronary CTA assessment time	Significant reduction	−27.0% (Not stated)
Baum et al.,^33^ 2023	USA	RCT; Single centre; Hospital	37^a^	NR	NR	Not applicable	AI-guided POCUS device	Apical four-chamber view image acquisition time	Significant reduction	−28 s (Not stated)
Chen et al.,^51^ 2021	China	RCT; Single centre; Hospital	80	36.3; 63.7	NR	Acute left heart failure	AI-enabled echocardiography	Diagnostic accuracy	No significant effect	Not stated
De Backer et al.,^34^ 2023	Belgium, Canada, Denmark, France, Italy, Sweden	RCT; Multicentre; Hospital	181	30.5; 69.5	NR	Nonvalvular atrial fibrillation	AI-enabled CT computational modelling for planning of LAA closure	Incomplete LAA closure with residual contrast leakage into the LAA distal of the Amulet lobe and/or presence of a device-related thrombus	No significant effect	RR 0.69 (0.46–1.04)
Deisenhofer et al.,^35^ 2025	Belgium, France, Germany, Netherlands, USA	RCT; Multicentre; Hospital	357	21.0; 79.0	NR	Persistent atrial fibrillation	AI-guided ablation of spatio-temporal dispersion areas plus PVI	AF recurrence at 12 months	Demonstrated superiority	HR 0.30 (0.21–0.57)
He et al.,^36^ 2023	USA	RCT; Single centre; Hospital	3035	43.0; 57.0	White (58.0)	Left ventricular ejection fraction	AI assessment of transthoracic echocardiogram	Change in LVEF between assessments	Demonstrated non-inferiority	−10.4% (proportion of change; −13.2 to −7.7)
Hill et al.,^37^ 2022	UK	RCT; Multicentre; Primary care practice	1880	45.3; 54.7	Unknown (68.9)	Undiagnosed atrial fibrillation	AI risk prediction algorithm plus diagnostic testing	Proportion of diagnoses of AF and related arrhythmias	No significant effect	OR 1.15 (0.77–1.73)
Horne et al.,^38^ 2022	USA	RCT; Multicentre; Hospital outpatient service	182	25.8; 74.2	White (98.4)	Statin indication	AI behaviour nudges	Proportion of days covered by statin prescription	Significant improvement	PDC 0.103 (Not stated)
Huang et al.,^39^ 2021	China	RCT; Single centre; Hospital	218	29.0; 71.0	NR	Non-valvular atrial fibrillation recurrence post-ablation	AI-enabled handheld single-lead ECG monitor	Detection of AF recurrence	Significant improvement	Not stated
Kelshiker et al.,^55^ 2026	UK	Cluster RCT; Multicentre	1,553,175	48.4; 61.6	White (41.2)	Reduced left ventricular ejection fraction (≤40%), atrial fibrillation, and valvular heart disease	Training and implementation in use of AI-stethoscopes in routine care	Incidence of newly coded diagnoses of heart failure	No significant effect	IRR 0.94 (0·86–1·02)
Kim et al.,^40^ 2025	South Korea	RCT; Multicentre; Hospital	395	18.2; 81.8	NR	Severe coronary artery disease	AI quantitative coronary angiography–assisted PCI	Post-PCI minimal stent area	Demonstrated non-inferiority	−0.16 mm^2^ (−0.59 to 0.28)
Kumar et al.,^56^ 2025	USA	RCT; Single centre; Hospital	38^a^	45.5; 54.5	NR	Not applicable	POCUS device with integrated DL-functionality	Total acquisition time for a comprehensive five-view limited echocardiogram	Significant reduction	Median acquisition time, 151 s (with AI; IQR: 115–195) versus 267 s (without AI; IQR: 206–325)
Labovitz et al.,^52^ 2017	USA	RCT; Single centre; Hospital outpatient service	27	53.6; 46.4	Black (46.0)	Ischaemic stroke	AI daily monitoring	Mean cumulative medication adherence	Significant improvement	50.0% (Not stated)
Lin et al.,^41^ 2024a	Taiwan	RCT; Multicentre; Hospital	43,234	50.5; 49.5	NR	ST-elevation myocardial infarction	AI-ECG assistance	Door-to-balloon time	Significant reduction	−14.00 min (Not stated)
Lin et al.,^42^ 2024b	Taiwan	RCT; Multicentre; Hospital	15,965	48.4; 51.6	NR	Vulnerable hospitalised patients	AI-ECG assistance	All-cause mortality within 90 days	Significant reduction	HR 0.83 (0.70–0.99)
Liu et al.,^43^ 2025	Taiwan	Cluster RCT; Multicentre; Hospital	520	46.7; 53.3	NR	Atrial fibrillation	AI-ECG assistance	New AF diagnosis; non–vitamin K antagonist oral anticoagulant (NOACs) prescription within 90 days after discharge; echocardiogram arrangement; cardiologist visits	Significant improvement on NOACs prescription and new AF diagnoses; No significant effect on echocardiogram arrangements and cardiologist visits.	HRs: NOACs, 1.85 (1.11–3.07), New AF diagnosis, 1.40 (1.03–1.90), Echocardiogram arrangements, 1.14 (0.90–1.44), Cardiologist visits, 1.42 (0.98–2.05).
Martinez-Gutierrez et al.,^44^ 2023	USA	Stepped-wedge, cluster RCT; Multicentre; Hospital	243	50.0; 50.0	White (44.0)	Large vessel occlusion acute ischaemic stroke	AI-enabled CT angiogram paired with mobile application	Door-to-groin time	Significant reduction	−11.20 min (−18.22 to −4.2)
O'Sullivan et al.,^57^ 2026	USA	RCT; Single centre; Hospital	107	NR	NR	Suspected genetic cardiomyopathy	LLM-assisted clinical assessment (AMIE system)	Subspecialist preference for clinical assessment quality	Significant difference	Preference percentage, 46.7% (with AI assistance) vs 32.7% (without AI assistance)
Persell et al.,^53^ 2020	USA	RCT; Multicentre; Primary care practice	297	61.3; 28.7	White (51.5)	Hypertension	Conversational artificial intelligence smartphone app plus home blood pressure monitor	Systolic blood pressure at 6 months	No significant effect	−2.0 mm Hg (−4.9 to 0.8)
Qian et al.,^45^ 2025	China	RCT; Single centre; Hospital	455	42.1; 57.9	NR	Suspected coronary artery disease	On-site deep learning-based CT-derived fractional flow reserve (CT-FFR)	ICA efficiency (rate of ICA with nonobstructive disease [stenosis <50%] and the ratio of revascularization to ICA [REV-to-ICA ratio] within 90 days)	Significant improvement	Risk difference: ICA with nonobstructive disease, −19.3% (−36.7% to −1.8%); REV-to-ICA ratio, 22.6% (2.5%–42.6%)
Sakamoto et al.,^58^ 2026	Japan	Randomised crossover; Single centre; Hospital	585	57.3; 42.7	NR	Screening echocardiography for preoperative assessments, chest pain evaluations, and for patients without a known history of cardiovascular disease	AI-assisted echocardiographic workflow	Efficiency of echocardiographic examinations (total time required per patient and total number of examinations per sonographer per day)	Significant improvement	Average examination time, −1.3 min (−0.68 to −1.93); Number of echocardiography scans per sonographer, 2.6 (2.19–3.01)
Sato et al.,^59^ 2025	Japan	RCT; Single centre; Home-based	289	9.7; 90.3	NR	Elevated blood pressure and sodium-sensitive AGT M235T genotype	Sodium reduction program (genetic profile + AI-based app + educational information)	Daily salt intake estimated from spot urine samples	No significant effect	−0.2 g/d (−0.7 to 0.3)
Trivedi et al.,^46^ 2025	Australia	RCT; Single centre; Hospital inpatient and outpatient services	84	30.1; 69.9	White (54.5)	Atrial fibrillation	Conversational AI phone calls	Atrial fibrillation specific quality of life (Atrial Fibrillation Effect on Quality-of-Life, AFEQT)	No significant effect	2.08 (−7.79 to 11.96)
Tsai et al.,^47^ 2025	Taiwan	RCT; Single centre; Hospital	13,631	54.6; 55.4	NR	Low ejection fraction (≤50%)	AI-ECG assistance	New diagnosis of low EF (≤50%) within 30 days of ECG	Significant improvement	HR 1.50 (1.11–2.03)
Upton et al.,^48^ 2024	UK	RCT; Multicentre; Hospital	2213	45.0; 55.0	White (71.5)	Coronary artery disease	AI-augmented stress echocardiogram	Appropriate referral for coronary angiography	Did not demonstrate non-inferiority	AUROC 0.09 (−0.22 to 0.39)
Wang et al.,^54^ 2019	USA	Stepped-wedge, cluster RCT; Multicentre; Primary care practice	1727	NR	NR	Atrial fibrillation	AI screening of EHR	New anticoagulant prescriptions	No significant effect	0.1% (Not stated)
Yang et al.,^49^ 2023	China	RCT; Multicentre; Hospital	1216	35.4; 64.6	NR	Stable coronary artery disease	On-site computed tomography–derived fractional flow reserve (CT-FFR) using machine learning	Unnecessary or delayed invasive coronary angiography	Significant reduction	−17.9% (Not stated)
Yao et al.,^60^ 2026	China	RCT; Multicentre; Home-based	62	41.9; 58.1	NR	Hypertension	AI-ECG assistance	Change in 6-min walk test distance	Significant improvement	Adjusted mean difference 62.77 (26.33–99.22)
Yao et al.,^50^ 2021	USA	Cluster RCT; Multicentre; Primary care practice	22,641	53.9; 46.1	White (94.3)	Asymptomatic left ventricular systolic dysfunction	AI-assisted remote rehabilitation plus routine health education	New diagnosis of low EF (≤50%) within 90 days of the ECG.	Significant improvement	OR 1.32 (1.08–1.61)
Zhang et al.,^61^ 2026	China	Cluster RCT; Multicentre; Hospitals	21,603	35.5; 64.5	NR	Acute ischaemic stroke	AI-based cerebrovascular disease clinical decision support system (CDSS)	New vascular event (composite of ischaemic stroke, haemorrhagic stroke, myocardial infarction, and vascular death) within three months after initial symptom onset.	Significant reduction	Adjusted HR 0.74 (0.58–0.93)

USA = United States of America, UK = United Kingdom, NR = not reported, RCT = randomised controlled trials, ICA = invasive coronary angiography, OR = odds ratio, RR = risk ratio, HR = hazard ratio, CI = confidence interval, EF = ejection fraction, AF = atrial fibrillation, ECG = electrocardiogram.

a Health professionals.

Trials were primarily funded by academic institutions (14 [45%]), followed by industry (7 [23%]) and mixed funding sources (5 [16%]). One trial was funded by a hybrid research entity (private-public partnership),^46^ two did not disclose the funding source,^39^^,^^51^ and two declared no funding received.^33^^,^^56^ The vast majority (27 [87%]) were two-arm, parallel-group trials. Only two trials employed a stepped-wedge, cluster randomised design.^44^^,^^54^ Of the parallel-group trials, 22 (81%) performed randomisation at the individual level and 5 (19%) at the cluster level. Most trials did not specify a hypothesis framework, although two described themselves as non-inferiority trials,^36^^,^^40^^,^^48^ one as a superiority trial,^35^ and five as pragmatic trials.^31^^,^^43^^,^^47^^,^^50^^,^^61^

Twenty-seven (87%) trials specified follow-up durations. Among these, median length was six months (range, 0.5–24; IQR, 3–12), and approximately one-third (11 [35%]) had a follow-up period of 12 months or longer. The remaining four trials either did not state the follow-up duration,^32^ had varied follow-up length,^31^ or had no clinical follow-up.^36^

Of the 31 trials included, 29 recruited a total of 1,685,717 patients (median, 455; range, 27–1,553,175; IQR, 182–2213). The remaining two trials involved health professionals only.^33^^,^^56^ Thirty (97%) trials provided information on attrition, with median attrition rate of 1.3% (range, 0.0–18.4; IQR, 0.0–5.2). Twenty-eight (90%) trials reported mean or median participant age, with the median number being 63.2 years (range, 31.0–78.6; IQR, 59.5–65.8). Sex was reported in 28 (90%) trials, with median proportion of female participants being 44.0% (range, 9.7–100.0; IQR, 33.3–50.1). One trial had female participants only as it focused on pregnancy-related cardiomyopathies.^31^

Race and ethnicity were reported in 11 (35%) trials. Among these, White participants comprised the majority in eight trials, with a median percentage of 56.2 (range, 41.2–98.4; IQR, 49.6–77.2). Black participants were the majority in two (8%) trials,^31^^,^^52^ including one trial in Nigeria with 100% Black participants.^31^ In one trial, the majority of participants was recorded as unknown race or ethnicity.^37^ None of the 13 trials conducted in Asia reported race or ethnicity of participants.^39^, ^40^, ^41^, ^42^, ^43^^,^^45^^,^^47^^,^^49^^,^^51^^,^^58^, ^59^, ^60^, ^61^

Approximately two-third of trials (20 [64%]) were conducted in hospital settings, with the remaining in outpatient clinics (two [6%]), primary care practices (4 [13%]), and community settings (home-based; 4 [13%]). One trial recruited patients from both inpatient and outpatient services of a hospital.^46^ Regarding target conditions, 7 trials (23%) evaluated AI interventions for atrial fibrillation, 6 (19%) for coronary artery disease, 6 (19%) for heart failure, 3 (10%) for hypertension, and 3 (10%) for ischaemic stroke. Other target conditions included genetic cardiomyopathies,^57^ vulnerable hospitalised patients,^42^ and patients with statin indications.^38^ Three studies on AI-enabled echocardiography were not designed for a specific condition.^33^^,^^56^^,^^58^

The characteristics of AI systems evaluated in the 31 trials are summarised in Table S4 of Supplementary Material. These systems were designed to address a range of downstream healthcare tasks that can be broadly grouped into four categories, namely disease screening and diagnostic support (15 trials [48%]), procedural guidance (6 [19%]), disease self-management (6 [19%]), and clinical decision support and recommendations (4 [13%]).

The AI systems spanned multiple application domains and utilised diverse input data modalities. Computer vision was the most frequent application domain, with 16 (52%) systems trained on images or video recordings derived primarily from clinical imaging tests such as echocardiography,^36^^,^^48^^,^^51^^,^^58^ computed tomography (CT) angiography,^32^^,^^44^^,^^45^^,^^49^ cardiac CT,^34^ coronary angiography,^40^ and point-of-care ultrasound (POCUS).^33^^,^^56^ Two systems were trained to analyse data from real-time smartphone camera video frames,^52^^,^^60^ one from meals images, and one from brain magnetic resonance imaging data. Signal analysis was reported in 9 (29%) trials, mainly on physiological waveforms from 12-lead or single-lead electrocardiographic recordings.^31^^,^^35^^,^^39^^,^^41^, ^42^, ^43^^,^^47^^,^^50^^,^^55^ Four (13%) models were trained on structured data from sources such as electronic health records and questionnaires.^37^^,^^38^^,^^54^^,^^57^ Two models (8%) used unstructured patient-generated data,^46^^,^^53^ one of which also incorporated automated speech recognition of participants’ vocal inputs.^46^

Most trials investigated deep-learning approaches, with artificial neural networks being the most commonly applied (19 [61%]). Of these, 16 trials evaluated convolutional neural networks (CNNs), either as standalone models (14 [88%]) or hybrid approaches (2 [12%]), such as CNN combined with bidirectional long short-term memory and CNN integrated with eXtreme Gradient Boosting.^35^^,^^39^ Other approaches included multilayer neural networks with bidirectional recurrent neural networks,^49^ time-varying neural networks,^37^ and reinforcement learning.^38^ Only one trial evaluated large language model.^57^ Notably, 10 (32%) trials did not specify the underlying AI algorithm or architecture, referring only to the use of “AI” without elaborating on technical detail. Even where algorithms were described, reporting was inconsistent, with few trials providing adequate methodological information or references to publications describing model development. The provenance of evaluated AI models was predominantly industry-based (21 [68%]), with the remainder developed in academic settings (10 [32%]).

The evaluated AI systems generated outputs directed toward care providers (25 [84%] trials) or patients (5 [16%]). The most common output type was regression or classification probabilities (14 [45%]), including percentile scores, binary disease classifications, disease risk scores, and continuous predictions such as ejection fraction or fractional flow reserve values. Seven (23%) systems provided anatomical structures labelling and quantitative measurements for care providers. They included coronary segment labelling and lesion quantification,^32^^,^^40^ real-time POCUS probe guidance,^33^^,^^56^ and visual and audio cues to guide ablation.^35^ Real-time alerts and notifications, often accompanied by regression or classification probabilities, were delivered to care providers in 6 (29%) systems via mobile applications or text messages to support timely clinical decision-making.^31^^,^^41^, ^42^, ^43^, ^44^^,^^46^ Six (29%) systems generated patient-facing outputs focused on health education and behavioural nudges in the form of automated text messages and emails, in-app conversations, interactive voice response calls, and exercise guidance.^38^^,^^46^^,^^52^^,^^53^^,^^59^^,^^60^ One system generated simulations of different Amulet device sizes and implant positions to support planning of left atrial appendage closure.^34^ System output formats in 3 (13%) trials were unclear.^36^^,^^39^^,^^54^

**Fig. 2:**
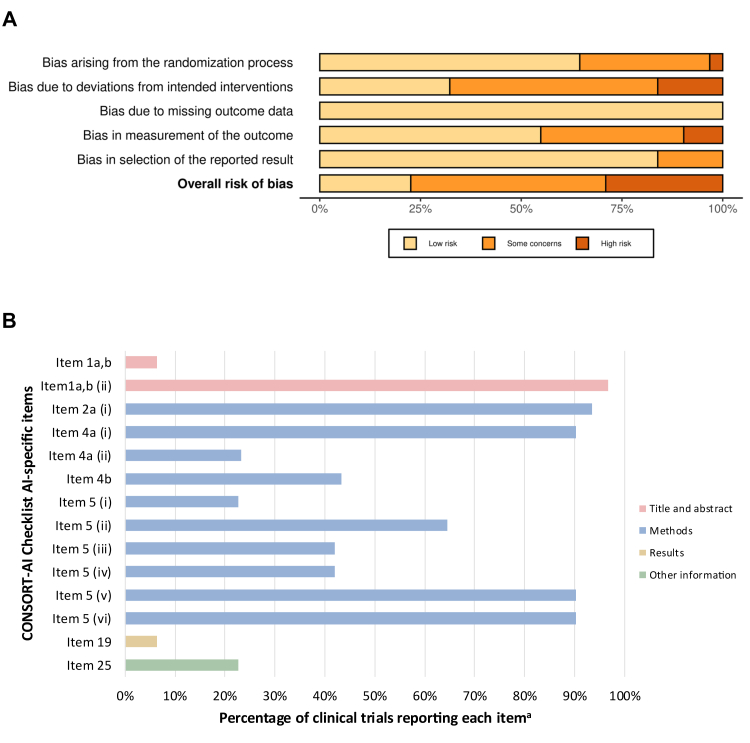
**A. Bar charts∗ showing the proportion of included trials rated as low risk of bias, some concerns, or high risk of bias for each domain; B. Reporting frequency for each of the 14 AI-specific items in CONSORT-AI Extension.**^21^ ∗Figure generated using robvis, Risk-of-bias visualisation. ^a^Calculated as (yes + not applicable)/(total number of trials). CONSORT-AI = Consolidated Standards of Reporting Trials-Artificial Intelligence. AI = artificial intelligence.

Results of risk of bias assessment are presented in Figure S2, Supplementary Material. Overall, 7 (23%) trials had a low risk of bias, 15 (48%) had some concerns, and 9 (29%) had a high risk of bias. The major potential sources of bias were risks from outcome measurements and deviations from intended interventions due to unmasking of trial participants and care providers (Fig. 2A). Due to the nature of AI interventions, the majority of trials adopted an open-label design. Trial registration number was reported in 27 (87%) trials, including one with retrospective registration.^33^ Four studies did not provide trial registration number and could not be identified by manual searching on clinical trial registries.^32^^,^^39^^,^^45^^,^^51^

Excluding three trials^52^, ^53^, ^54^ that were published before the introduction of CONSORT-AI extension in September 2020,^21^ fewer than one-third (8 [26%]) of eligible trials explicitly cited the CONSORT-AI reporting guidelines.^31^^,^^36^^,^^42^^,^^43^^,^^47^^,^^50^^,^^55^^,^^61^ Overall trial adherence to the 14 AI-specific items of the checklist was moderate (median, 57% [range, 21–86; IQR, 43–64]). No trial reported all 14 AI-specific components. The most frequent shortcomings were omission of model type in the abstract, omission of AI algorithm version used, inadequate reporting on inclusion and exclusion criteria for input data, lack of performance errors analysis, and absence of code or algorithm availability (Fig. 2B).

A wide range of study endpoints were found across the 31 included trials (Table 1). All but one trial^43^ had a single primary endpoint. The exception is an AI-enabled ECG alert study for atrial fibrillation, which specified four primary endpoints: new-onset AF diagnosis, oral anticoagulant prescription, echocardiogram arrangement, and cardiologist visits.^43^ Primary endpoints were related to clinical decision-making in seven (23%) trials, process of care and service utilisation in eight (26%), diagnostic and prognostic performance in three (10%), and patient's health and behaviour in 12 trials (39%). The remaining one trial assessed subspecialist preference for clinical assessment quality between AI-assisted and non-AI assisted cardiologists.^57^ Only three trials used hard clinical endpoints, namely all-cause mortality, new vascular event, and disease recurrence, as their primary endpoints.^35^^,^^42^^,^^61^

Overall, over two-thirds (22 [71%]) of the trials reported significant effects of AI intervention on their respective primary endpoints,^31^, ^32^, ^33^^,^^35^^,^^36^^,^^38^, ^39^, ^40^, ^41^, ^42^, ^43^, ^44^, ^45^^,^^47^^,^^49^^,^^50^^,^^52^^,^^56^, ^57^, ^58^^,^^60^^,^^61^ the majority of which were non-hard clinical outcomes. AI was found to have no significant effect on the primary endpoints in nine (29%) trials.^34^^,^^37^^,^^46^^,^^51^^,^^53^, ^54^, ^55^^,^^59^ Among the four trials with pre-specified hypotheses, two demonstrated non-inferiority of AI-assisted cardiovascular care to routine care,^36^^,^^40^ one could not establish the non-inferiority of AI-augmented stress echocardiogram compared to standard clinical decision-making,^48^ and one showed that AI-guided ablation of spatio-temporal dispersion areas plus pulmonary vein isolation (PVI) was superior to PVI alone.^35^ Six trials assessed procedural time as primary outcomes, all of which reported significant reductions with AI interventions.^32^^,^^33^^,^^41^^,^^44^^,^^56^^,^^58^

Eleven trials^31^^,^^38^^,^^45^^,^^47^^,^^49^^,^^50^^,^^53^^,^^55^^,^^59^, ^60^, ^61^ (n = 1,614,689 participants) were included in the meta-analyses, of which two were judged to have high risk of bias, seven with some concerns, and two with low risk of bias. The quality of evidence ranged from low to very low across outcomes due to risk of bias, indirectness, and imprecision (Table 2).

**Table 2:** Summary of findings.

Outcomes (no. of studies)	No. of patients		Relative effect (95% CI)	Absolute effect^a^ 95% CI)	Certainty of the evidence (GRADE)^b^	Comments
	Intervention	Comparison				
Systolic BP (3 trials)	305	316	–	mean 3.18 mm Hg lower (5.5 lower to 0.86 lower)	⨁⨁◯◯ Low^c^^,^^d^	Intervention may result in reduction in systolic BP.
Diastolic BP (3 trials)	305	316	–	mean 1.3 mm Hg lower (3.96 lower to 1.37 higher)	⨁⨁◯◯ Low^c^^,^^d^	Intervention may result in little to no difference in diastolic BP.
BMI (2 trials)	274	285	–	mean 0.02 kg/m^2^ higher (0.52 lower to 0.57 higher)	⨁⨁◯◯ Low^c^^,^^e^	Intervention may result in little to no difference in BMI.
MACE (3 trials)	379/11,838 (3.2%)	494/11,380 (4.3%)	RR 0.74 (0.58–0.96)	11 fewer per 1000 (from 18 fewer to 2 fewer)	⨁⨁◯◯ Low^f^^.^^g^	Intervention may result in reduction in MACE.
HF diagnosis (4 trials)	1713/720,933 (0.2%)	2249/869,709 (0.3%)	RR 1.36 (1.00–1.85)	1 more per 1000 (from 1 fewer to 3 more)	⨁⨁◯◯ Low^c^^,^^e^	Intervention may result in little to no difference in HF diagnosis.
Echocardiogram utilisation (2 trials)	3392/18,413 (18.4%)	3192/17,859 (17.9%)	RR 1.03 (0.69–1.53)	5 more per 1000 (from 55 fewer to 95 more)	⨁◯◯◯ Very low^c^^,^^d^^,^^e^	Uncertain.
AF diagnosis (2 trials)	182/1181 (15.4%)	136/1192 (11.4%)	RR 1.27 (0.49–3.28)	31 more per 1000 (from 58 fewer to 260 more)	⨁◯◯◯ Very low^c^^,^^e^^,^^h^	Uncertain.

AF = atrial fibrillation, BMI = body mass index, BP = blood pressure, CI = confidence interval, HF = heart failure, MACE = major adverse cardiovascular events, RR = risk ratio.

a The risk in the intervention group (and its 95% CI) is based on the assumed risk in the comparison group and the relative effect of the intervention (and its 95% CI).

b GRADE Working Group grades of evidence.

c Some concerns with deviations from intended intervention and outcome measurements.

d Wide 95% confidence interval.

e Surrogate outcome.

f Some concerns with deviations from intended intervention and selective reporting.

g Wide 95% confidence interval that crosses 25% relative risk reduction effect.

h Optimal information size not met and wide 95% confidence interval.

**Fig. 3:**
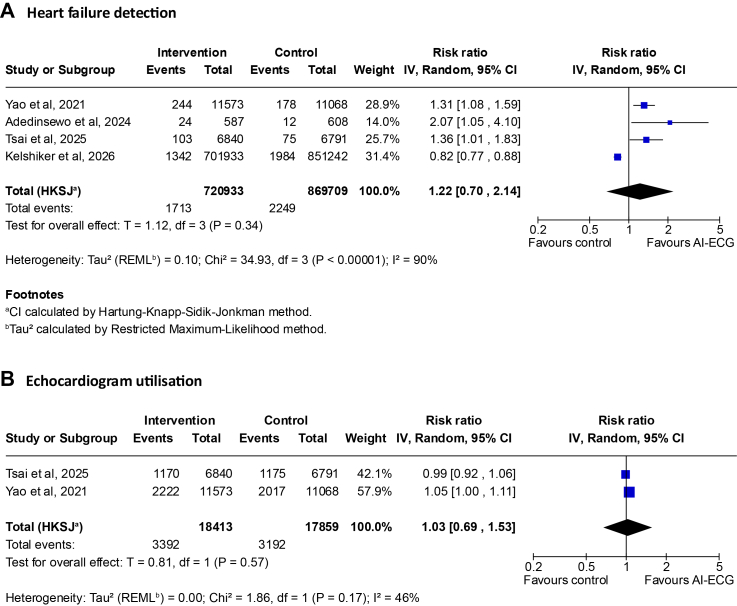
**Forest plots of (A) heart failure detection and (B) echocardiogram utilisation reported in studies on AI-ECG interventions for identifying heart failure.**^a^CI calculated by HKSJ method. ^b^Tau^2^ calculated by REML method. AI-ECG = artificial intelligence-enhanced electrocardiogram. HKSJ = Hartung-Knapp-Sidik-Jonkman. REML = Restricted Maximum-Likelihood method.

Four studies evaluated AI-enhanced ECGs for identifying patients with heart failure.^31^^,^^47^^,^^50^^,^^55^ AI-enhanced ECGs did not result in significant difference in heart failure detection (RR 1.22 [95% CI 0.70–2.14]; p = 0.34; 1,590,642 participants; Fig. 3A), with substantial heterogeneity observed among studies (χ^2^ = 34.93; p < 0.00001; I^2^ = 90%). Exclusion of study by Kelshiker et al.,^55^ which contributed an outlying effect size in the opposite direction and carried the greatest weight, reduced statistical heterogeneity substantially (χ^2^ = 1.60; p = 0.45; I^2^ = 0%) but did not change the direction of associations and statistical significance (RR 1.36 [95% CI 1.00–1.85]; p = 0.05). Sensitivity analysis excluding study with high risk of bias (Yao et al.,^50^ 2021) did not change the direction of association and statistical significance of pooled effect estimates. Certainty of evidence was ranked low due to risk of bias and indirectness (surrogate endpoint).

Two of the four trials on AI-enhanced ECG also^47^^,^^50^ reported on echocardiogram utilisation for confirmation of low EF following initial AI-ECG screening. It is uncertain whether AI was associated with any difference in echocardiogram utilisation (RR 1.03 [95% CI 0.69–1.53]; p = 0.57; 36,272 participants; Fig. 3B; very low certainty). There was moderate heterogeneity observed (χ^2^ = 1.86; p = 0.17; I^2^ = 46%). Certainty of evidence was downgraded to very low due to risk of bias, indirectness (surrogate endpoint), and imprecision (wide 95% CI).

**Fig. 4:**
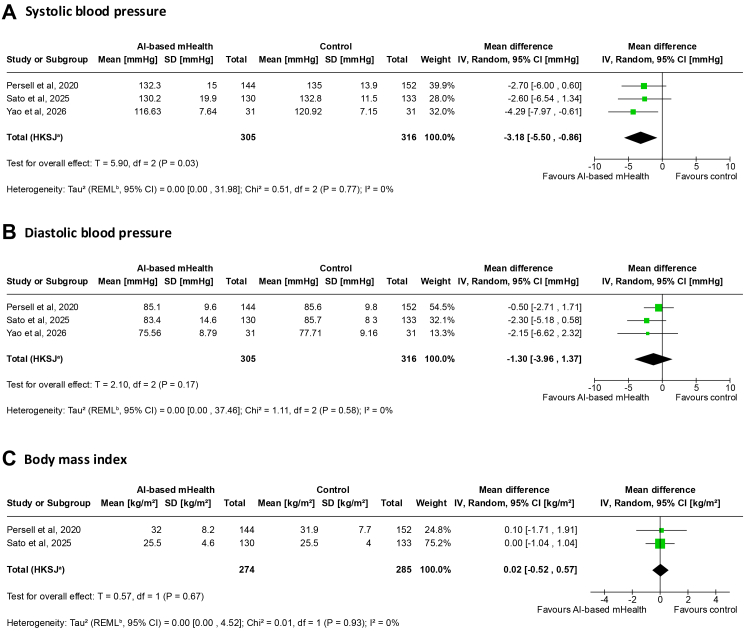
**Forest plots of (A) systolic blood pressure (mm Hg), (B) diastolic blood pressure (mm Hg), and (C) body mass index (kg/m^2^) reported in studies on AI-based mobile health (mHealth) interventions for hypertension self-management.**^a^CI calculated by KHSJ method. ^b^Tau^2^ calculated by REML method. AI = artificial intelligence. mHealth = mobile health. HKSJ = Hartung-Knapp-Sidik-Jonkman. REML = Restricted Maximum-Likelihood method.

Three trials^53^^,^^59^^,^^60^ on AI-based mobile health app (mHealth) for hypertension self-management reported blood pressure (BP) outcomes. AI-based mHealth interventions were associated with significant reduction in systolic BP (mean difference −3.18 mm Hg [95% CI −5.50 to −0.86]; p = 0.03; 621 participants; Fig. 4A; low certainty), with no evidence of statistical heterogeneity (χ^2^ = 0.51; p = 0.77; I^2^ = 0%). However, no statistically significant effect on diastolic BP was observed (mean difference −1.30 mm Hg [95% CI −3.96 to 1.37]; p = 0.17; I^2^ = 0%; 621 participants; Fig. 4B; low certainty). Two trials^53^^,^^59^ reported on body mass index (BMI) and showed no significant effect demonstrated by AI-based mHealth (mean difference −0.02 kg/m^2^ [95% CI −0.52 to 0.57]; p = 0.67; 559 participants; Fig. 4C; low certainty), with no evidence of statistical heterogeneity (χ^2^ = 0.1; p = 0.93; I^2^ = 0%). Certainty of evidence was low for systolic and diastolic BP due to risk of bias and imprecision (wide 95% CI), and low for BMI due to risk of bias and indirectness (surrogate outcome).

**Fig. 5:**
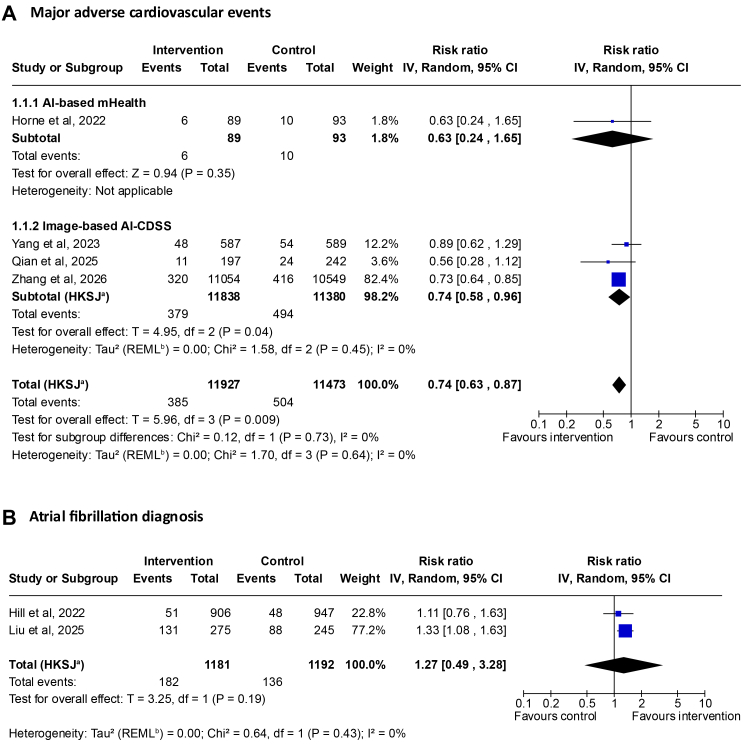
**Forest plots of (A) major adverse cardiovascular events and (B) atrial fibrillation diagnosis.**^a^CI calculated by HKSJ method. ^b^Tau^2^ calculated by REML method. AI = artificial intelligence. mHealth = mobile health. CDSS = clinical decision support system. HKSJ = Hartung-Knapp-Sidik-Jonkman. REML = Restricted Maximum-Likelihood method.

Major adverse cardiovascular events (MACE) were reported in four studies,^38^^,^^45^^,^^49^^,^^61^ including three on image-based AI-CDSS. MACE definitions varied slightly across studies but consistently comprised major cardiovascular events, including myocardial infarction, revascularization, hospitalisations for acute coronary syndrome, and mortality. Image-based AI-CDSS was associated with significantly lower MACE (RR 0.74 [95% CI 0.58–0.96]; p = 0.04; 3 trials; 23,218 participants; Fig. 5A). There was no evidence of statistical heterogeneity (χ^2^ = 1.58; p = 0.45; I^2^ = 0%). However, leave-one-out analysis indicated that the pooled estimate was sensitive to individual studies, as exclusion of any single study resulted in loss of statistical significance (Expanded Method S3). Certainty of evidence was ranked low due to risk of bias and imprecision (wide 95% CI that crosses 25% relative risk reduction effect).

Two studies^37^^,^^43^ reported the diagnosis of AF among previously undiagnosed individuals. It is uncertain whether AI was associated with any difference in AF diagnosis (RR 1.27 [95% CI 0.49–3.28]; p = 0.19; 2373 participants; Fig. 5B; very low certainty), with no evidence of significant statistical heterogeneity (χ^2^ = 0.64; p = 0.43; I^2^ = 0%). Quality of evidence was ranked very low due to risk of bias, indirectness (surrogate endpoint), and imprecision (optimal information size not met and wide 95% CI).

Five studies^38^^,^^39^^,^^46^^,^^52^^,^^53^ (n = 625 participants) on AI-enhanced disease self-management systems reported medication adherence as dichotomous outcome (adherent versus non-adherent participants). Due to the heterogeneity in ascertainment of medication adherence (self-reported medication adherence, proportion of days covered, and plasma drug concentration levels) and the types of medication assessed, meta-analysis was deemed inappropriate and results were instead summarised using vote-counting (Table S5). The evidence was mixed: two studies reported statistically significant improvement in proportion of adherent participants with AI interventions,^38^^,^^39^ two found no significant effect,^46^^,^^53^ and one reported higher proportion of adherent participants in the AI group but did not test for between-group differences.^52^

Detailed results of sensitivity analyses to assess the robustness of the meta-analytic effect estimates are described in Expanded Method S3. Overall, the effect estimates for MACE, systolic BP, and diastolic BP were sensitive to individual studies, as removal of single study resulted in either gain or loss of statistical significance. Effect estimate for HF diagnosis was not sensitive to individual studies. Leave-one-out analysis was not performed for echocardiogram utilisation, BMI, and AF diagnosis, as only two studies contributed to meta-analysis of these outcomes. For sensitivity to analytical approaches, the summary effect of HF diagnosis became statistically significant when estimated using fixed-effect model and random-effect model with Wald-type method. Effect estimates for other outcomes were not sensitive to analytical approaches. Funnel plots were generated for visualisation purposes (Figure S3).

## Discussion

In this systematic review and meta-analysis, we identified 31 trials evaluating AI in cardiovascular care published between 2017 and 2025. Most trials were published from 2021 onwards, single-country, individually randomised, hospital-based, and had short follow-up periods. Over two-thirds reported statistically significant effects of AI on their primary endpoints, which were mostly intermediate process measures, although consistent evidence across disease areas, care settings, and clinically meaningful outcomes remains limited. Trial endpoints were heterogeneous, spanning clinical decision-making, processes of care, diagnostic performance, and patient behaviour. AI systems were predominantly designed to support disease detection and diagnostic tasks, frequently employing computer vision or signal analysis based on deep-learning architectures such as artificial neural networks. Only a quarter of trials were judged to be at low risk of bias, and overall adherence of trial reporting to AI-specific items in CONSORT-AI was moderate. Meta-analyses showed statistically significant effect of AI interventions on MACE and systolic blood pressure, but not on other outcomes. The certainty of evidence was low or very low across all outcomes. Collectively, the evidence reflects that strengthened methodological rigour, standardisation, and transparency in reporting are required to fulfil the potential of AI in clinical translation.

The geographical distribution of cardiovascular AI trials has been highly uneven. Despite its increasing number since 2021, we found a dominance of trials conducted in high-income countries, especially from the USA and European countries. Similar geographical concentration was observed in reviews of RCTs on AI in broader healthcare.^13^, ^14^, ^15^ Notably, fewer than a quarter of trials in this review were conducted in low and middle-income countries (LMICs), despite these regions carrying a substantial share of the global CVD burden.^62^ The paucity of evidence from LMICs limits the generalisability of findings and risks widening existing disparities, as the populations likely to benefit the most from AI-enabled cardiovascular care remain underrepresented in clinical evaluation. Contrary to the tendency of healthcare AI trials to be single-centre with small sample size,^15^ this review shows a relatively high proportion of multicentre cardiovascular AI trials, including several large, pragmatic trials enrolling more than 10,000 participants.

CVD trials in general are prone to non-representative enrolment, with females and ethnic minority groups frequently under-represented relative to their burden of disease in the population.^63^ This imbalance extends to cardiovascular AI trials. In this review, race or ethnicity was not reported in over half of the trials, and where reported, ethnic-minority groups were under-represented in the enrolled cohorts. These findings are consistent with prior reviews of RCTs on machine learning interventions in healthcare.^14^^,^^15^ Such gaps could undermine the external validity of trial results and impede assessment of the extent to which AI performance varies across population subgroups. This is a pertinent consideration for AI implementation in healthcare, as algorithmic racial bias in clinical AI is well-documented.^64^ Without deliberate mitigation, such bias can lead to substandard care for ethnic-minority groups and exacerbate existing health disparities.^65^^,^^66^ Notwithstanding, we did not find evidence suggesting systematic under-enrolment of female participants in cardiovascular AI trials. Although none of the 11 trials conducted in Asia reported race and ethnicity, this likely reflects the relative demographic homogeneity of the four East Asian regions (China, South Korea, Japan, and Taiwan) studied. Nonetheless, transparent demographic reporting remains essential to enable evaluation of algorithmic fairness and risk of performance degradation in under-represented groups in order to ensure the equitable evaluation and deployment of AI systems.

AI interventions were evaluated against a plurality of primary endpoints. Over two-thirds of the included trials demonstrated statistically significant benefit of AI on primary endpoints, but most were intermediate surrogate outcomes related to clinical decision-making, process of care, service utilisation, and clinical biomarkers. Although these outcomes are more cost and time-saving to assess, gains in surrogate measures such as increased test ordering, higher diagnostic yield, and improved medication adherence do not necessarily translate into better patient health outcomes.^67^ Furthermore, modifications to care pathways can inadvertently result in additional investigations and visits, with increased costs and patient burden without net clinical benefit.^68^ This is supported by the results of our meta-analyses, where most evidence relates to intermediate process measures or surrogate outcomes, with limited evidence on hard clinical endpoints. The absence of mediation analyses in these trials also restricts understanding of the causal pathways through which AI-enabled changes in processes of care might influence downstream clinical outcomes. More rigorous and adequately powered trials with clinically meaningful endpoints are required to establish whether AI confers tangible benefits for patients beyond improvements in intermediate processes of care.

The current evidence base reveals condition-specific gaps that highlight priorities for future AI trials in cardiovascular care. In hypertension, trials with longer follow-up are needed to determine whether AI-supported self-management produces sustained blood pressure control and reduces downstream cardiovascular events. In coronary artery disease, trials should move beyond workflow efficiency to assess the impact of AI-guided imaging or decision support on hard clinical outcomes such as MACE and mortality. Likewise, future trials of AI-enhanced ECG for heart failure screening should establish whether earlier detection translates into earlier treatment and better health outcomes.

Several implementation considerations temper the translational relevance of the current evidence base. Most included trials were conducted in controlled or academic settings, which may not reflect real-world implementation. Clinician training requirements and integration into existing clinical workflows were rarely described, despite being critical determinants of real-world effectiveness. Similarly, none of the included studies addressed data drift and performance degradation,^69^ representing a key gap for sustained deployment of medical AI systems.

Our review highlights concerns regarding risk of bias, transparency, and reproducibility in trials of AI-enabled cardiovascular care. Three-quarters of included trials were judged at overall high risk of bias or with some concerns, most commonly due to shortcomings in outcome measurement and deviation from intended interventions, raising questions about the reliability of observed effects. Transparency in trial reporting was particularly limited. Not only was the explicit reference to CONSORT-AI extension guideline uncommon, overall concordance of trial with AI-specific items was moderate. These were also noted in previous studies that examined RCTs of AI interventions.^14^^,^^70^ Such gaps in methodological rigour and reporting standards could hinder reproducibility, limit external validity, and impede the safe and responsible translation of AI technologies into cardiovascular practice.

This systematic review has several limitations that warrant cautious interpretation of the findings. First, although we did not apply language filters in the electronic search, our eligibility criterion of English-language publications potentially excluded relevant trials published in other languages. Prior evidence suggests that investigators in non-English-speaking countries are more likely to publish negative findings in local language journals, and excluding these studies could introduce language bias.^71^^,^^72^ This concern is particularly salient for AI-related trials, as a recent analysis showed that four of the five countries with the highest number of registered clinical trials for AI or ML-enabled medical devices are non-English-speaking.^12^ Second, despite a comprehensive search across multiple databases and trial registries, unpublished or ongoing trials were likely missed, introducing potential publication and time-lag bias. For instance, we identified multiple relevant RCTs that were published as preprint articles at the time of study selection.^73^^,^^74^

Third, despite the large overall sample size, the evidence base is dominated by a small number of large pragmatic trials, resulting in disproportionate weighting of individual studies in several meta-analyses. Consequently, some pooled estimates may primarily reflect the findings of single trials, limiting their robustness and generalisability. Furthermore, the small number of contributing studies for several outcomes reduces the effective statistical power, despite the aggregate sample size. These limitations should be considered when interpreting the findings. Fourth, the rapid evolution of AI technologies means that some evaluated systems may no longer reflect current practice, and the findings should be interpreted within this context. We anticipate that this systematic review needs updating in the next few years, given the rapid pace of AI innovation.

Our findings highlight the rapid expansion of AI applications in cardiovascular care but also reveals gaps in methodological rigour and reporting transparency. Effects of AI interventions were inconsistent across disease areas and clinical settings, and the overall risk of bias was high. While AI may have potential benefits in intermediate process measures in cardiovascular care, low to very-low certainty evidence suggests current applications had unclear effect on patient health outcomes. Trial reporting frequently lacked transparency, with moderate adherence to CONSORT-AI standards, limited disclosure of algorithms, and incomplete demographic data, particularly regarding race and ethnicity. Evidence on AI-enabled cardiovascular care remains concentrated in high-income countries, with underrepresentation of low- and middle-income regions where CVD burden is greatest. More rigorous, inclusive, and transparently reported RCTs with clinically meaningful endpoints are needed to establish when and how AI can be safely and effectively integrated into cardiovascular practice.

## Contributors

AQCO conceived this systematic review. AQCO developed the search strategy and study protocol with CSA, JG, and NML. AQCO, CSA, IB, CLJ, and NML contributed to data extraction and analysis. JG and NML provided methodological guidance and supervised this review. AQCO wrote the first draft of this manuscript. CSA, IB, CLJ, HA, FSN, JC, PL, JG, and NML interpreted data and revised the manuscript critically for important intellectual content. All authors reviewed and approved the final version of the manuscript. AQCO, CSA, and NML accessed and verified the underlying data.

## Data sharing statement

All data used in this review were derived from published primary studies. Data supporting the findings are available in the Supplementary Material and from the corresponding author upon request.

## Declaration of interests

AQCO is supported by Yayasan Khazanah and Oxford Centre for Islamic Studies through the Merdeka Scholarship. FSN is supported by the British Heart Foundation programme grant (RG/F/22/110078), the National Institute for Health Research Imperial Biomedical Research Centre, the UK Medical Research Council, the Academy of Medical Sciences, and the British Council. FSN receives speaker fees from GE Healthcare, holds an AI-ECG patent, serves on the advisory board of J&J Medtech and on the Heart Rhythm Society Research Committee, and is a co-founder and shareholder of Cardiovolt.ai, a company commercialising AI-ECG. PL receives grants and contracts from the UK Medical Research Council, the UK National Institute for Health and Social Care Research, the British Heart Foundation, Merck & Cie, Osler Diagnostics, Ultromics Ltd, and Novo Nordisk. PL receives consulting fees from Ultromics Ltd and speaker fees from Pfizer and Bracco (Medical Imaging). PL served on the advisory board of Daiichi Sankyo (lipid therapies, 2024) and AstraZeneca (resistant hypertension, 2025), as member of UK NICE Cardiovascular Prevention Guideline Committee (2021–2024), and serves as Chair of UK NIHR i4i Product Development Awards Funding Panel (since 2025). PL is a founder and shareholder of Ultromics, an AI imaging company, and is named inventor on granted patents related to AI and imaging. CSA, IB, CLJ, JC, JG, and NML declare no competing interests. The views expressed are those of the author(s) and do not necessarily represent the views of any funder or institution.
