# Supplementary material for: Artificial intelligence in cardiovascular care: a systematic review and meta-analysis of randomised controlled trials
Source: eClinicalMedicine. 2026 Aug 6;98:104123. doi: 10.1016/j.eclinm.2026.104123 (PMC13475654; doi:10.1016/j.eclinm.2026.104123)
Supplement: Supplementary file 1 [file mmc1.docx]

**Supplementary Material**

**Artificial Intelligence in Cardiovascular Care: A Systematic Review and Meta-analysis of Randomised Controlled Trials**

Table of Contents

[Table S1: PRISMA 2020 for Abstracts Checklist	2](#_Toc230970277)

[Table S2: PRISMA 2020 Checklist	3](#_Toc230970278)

[Expanded Method S1. Full search strategy	6](#_Toc230970279)

[Ovid MEDLINE	6](#_Toc230970280)

[Ovid Embase	9](#_Toc230970281)

[Scopus	11](#_Toc230970282)

[CENTRAL	12](#_Toc230970283)

[Clinicaltrials.gov	13](#_Toc230970284)

[Expanded Method S2. Eligibility criteria for study selection	14](#_Toc230970285)

[Table S3. List of excluded studies and reasons for exclusion	15](#_Toc230970286)

[Figure S1. Temporal and geographical distribution of RCTs	24](#_Toc230970287)

[Table S4. Characteristics of AI interventions	25](#_Toc230970288)

[Figure S2. Risk of bias of included trials	30](#_Toc230970289)

[Figure S3. Funnel plots	31](#_Toc230970290)

[Expanded Method S3. Sensitivity analyses	33](#_Toc230970291)

[Major adverse cardiovascular events (MACE)	33](#_Toc230970292)

[Systolic blood pressure	33](#_Toc230970293)

[Diastolic blood pressure	34](#_Toc230970294)

[Body mass index	34](#_Toc230970295)

[Heart failure diagnosis	34](#_Toc230970296)

[Echocardiogram utilisation	34](#_Toc230970297)

[Atrial fibrillation diagnosis	35](#_Toc230970298)

[Table S5. Vote-counting of medication adherence	36](#_Toc230970299)

[References	37](#_Toc230970300)

## **Table S1: PRISMA 2020 for Abstracts Checklist**

| **Section and Topic** | **Item #** | **Checklist item** | **Reported (Yes/No)** |
| --- | --- | --- | --- |
| **TITLE** | | |  |
| Title | 1 | Identify the report as a systematic review. | Yes |
| **BACKGROUND** | | |  |
| Objectives | 2 | Provide an explicit statement of the main objective(s) or question(s) the review addresses. | Yes |
| **METHODS** | | |  |
| Eligibility criteria | 3 | Specify the inclusion and exclusion criteria for the review. | Yes |
| Information sources | 4 | Specify the information sources (e.g. databases, registers) used to identify studies and the date when each was last searched. | Yes |
| Risk of bias | 5 | Specify the methods used to assess risk of bias in the included studies. | Yes |
| Synthesis of results | 6 | Specify the methods used to present and synthesise results. | Yes |
| **RESULTS** | | |  |
| Included studies | 7 | Give the total number of included studies and participants and summarise relevant characteristics of studies. | Yes |
| Synthesis of results | 8 | Present results for main outcomes, preferably indicating the number of included studies and participants for each. If meta-analysis was done, report the summary estimate and confidence/credible interval. If comparing groups, indicate the direction of the effect (i.e. which group is favoured). | Yes |
| **DISCUSSION** | | |  |
| Limitations of evidence | 9 | Provide a brief summary of the limitations of the evidence included in the review (e.g. study risk of bias, inconsistency and imprecision). | Yes |
| Interpretation | 10 | Provide a general interpretation of the results and important implications. | Yes |
| **OTHER** | | |  |
| Funding | 11 | Specify the primary source of funding for the review. | Yes |
| Registration | 12 | Provide the register name and registration number. | Yes |

*From:* Page MJ, McKenzie JE, Bossuyt PM, Boutron I, Hoffmann TC, Mulrow CD, et al. The PRISMA 2020 statement: an updated guideline for reporting systematic reviews. BMJ 2021;372:n71. doi: 10.1136/bmj.n71. This work is licensed under CC BY 4.0. To view a copy of this license, visit <https://creativecommons.org/licenses/by/4.0/>

## **Table S2: PRISMA 2020 Checklist**

| **Section and Topic** | **Item #** | **Checklist item** | **Location where item is reported** |
| --- | --- | --- | --- |
| **TITLE** | | |  |
| Title | 1 | Identify the report as a systematic review. | Title |
| **ABSTRACT** | | |  |
| Abstract | 2 | See the PRISMA 2020 for Abstracts checklist. | Abstract |
| **INTRODUCTION** | | |  |
| Rationale | 3 | Describe the rationale for the review in the context of existing knowledge. | Page 6 |
| Objectives | 4 | Provide an explicit statement of the objective(s) or question(s) the review addresses. | Page 6 |
| **METHODS** | | |  |
| Eligibility criteria | 5 | Specify the inclusion and exclusion criteria for the review and how studies were grouped for the syntheses. | Page 7; Expanded Method S2 |
| Information sources | 6 | Specify all databases, registers, websites, organisations, reference lists and other sources searched or consulted to identify studies. Specify the date when each source was last searched or consulted. | Page 7 |
| Search strategy | 7 | Present the full search strategies for all databases, registers and websites, including any filters and limits used. | Page 5; Expanded Method S1 |
| Selection process | 8 | Specify the methods used to decide whether a study met the inclusion criteria of the review, including how many reviewers screened each record and each report retrieved, whether they worked independently, and if applicable, details of automation tools used in the process. | Pages 7 |
| Data collection process | 9 | Specify the methods used to collect data from reports, including how many reviewers collected data from each report, whether they worked independently, any processes for obtaining or confirming data from study investigators, and if applicable, details of automation tools used in the process. | Pages 7 |
| Data items | 10a | List and define all outcomes for which data were sought. Specify whether all results that were compatible with each outcome domain in each study were sought (e.g. for all measures, time points, analyses), and if not, the methods used to decide which results to collect. | Pages 7-8 |
|  | 10b | List and define all other variables for which data were sought (e.g. participant and intervention characteristics, funding sources). Describe any assumptions made about any missing or unclear information. | Pages 7-8 |
| Study risk of bias assessment | 11 | Specify the methods used to assess risk of bias in the included studies, including details of the tool(s) used, how many reviewers assessed each study and whether they worked independently, and if applicable, details of automation tools used in the process. | Page 9-9 |
| Effect measures | 12 | Specify for each outcome the effect measure(s) (e.g. risk ratio, mean difference) used in the synthesis or presentation of results. | Page 7-8 |
| Synthesis methods | 13a | Describe the processes used to decide which studies were eligible for each synthesis (e.g. tabulating the study intervention characteristics and comparing against the planned groups for each synthesis (item #5)). | Pages 7-8 |
|  | 13b | Describe any methods required to prepare the data for presentation or synthesis, such as handling of missing summary statistics, or data conversions. | Page 7-8 |
|  | 13c | Describe any methods used to tabulate or visually display results of individual studies and syntheses. | Page 7-8 |
|  | 13d | Describe any methods used to synthesize results and provide a rationale for the choice(s). If meta-analysis was performed, describe the model(s), method(s) to identify the presence and extent of statistical heterogeneity, and software package(s) used. | Pages 7-8 |
|  | 13e | Describe any methods used to explore possible causes of heterogeneity among study results (e.g. subgroup analysis, meta-regression). | Pages 7-8 |
|  | 13f | Describe any sensitivity analyses conducted to assess robustness of the synthesized results. | Page 8 |
| Reporting bias assessment | 14 | Describe any methods used to assess risk of bias due to missing results in a synthesis (arising from reporting biases). | Page 8 |
| Certainty assessment | 15 | Describe any methods used to assess certainty (or confidence) in the body of evidence for an outcome. | Page 8-9 |
| **RESULTS** | | |  |
| Study selection | 16a | Describe the results of the search and selection process, from the number of records identified in the search to the number of studies included in the review, ideally using a flow diagram. | Page 9; Figure 1 |
|  | 16b | Cite studies that might appear to meet the inclusion criteria, but which were excluded, and explain why they were excluded. | Supplementary Table S3 |
| Study characteristics | 17 | Cite each included study and present its characteristics. | Table 1; Supplementary Table S4 |
| Risk of bias in studies | 18 | Present assessments of risk of bias for each included study. | Figure 2; Supplementary Figure S2 |
| Results of individual studies | 19 | For all outcomes, present, for each study: (a) summary statistics for each group (where appropriate) and (b) an effect estimate and its precision (e.g. confidence/credible interval), ideally using structured tables or plots. | Figures 3, 4, 5 |
| Results of syntheses | 20a | For each synthesis, briefly summarise the characteristics and risk of bias among contributing studies. | Pages 13-14 |
|  | 20b | Present results of all statistical syntheses conducted. If meta-analysis was done, present for each the summary estimate and its precision (e.g. confidence/credible interval) and measures of statistical heterogeneity. If comparing groups, describe the direction of the effect. | Pages 13-14 |
|  | 20c | Present results of all investigations of possible causes of heterogeneity among study results. | Pages 13-14; Expanded  Method 3 |
|  | 20d | Present results of all sensitivity analyses conducted to assess the robustness of the synthesized results. | Pages 13-14; Expanded  Method S3 |
| Reporting biases | 21 | Present assessments of risk of bias due to missing results (arising from reporting biases) for each synthesis assessed. | Figure 2; Supplementary Figure S2 |
| Certainty of evidence | 22 | Present assessments of certainty (or confidence) in the body of evidence for each outcome assessed. | Pages 13-14; Table 2 |
| **DISCUSSION** | | |  |
| Discussion | 23a | Provide a general interpretation of the results in the context of other evidence. | Pages 14-16 |
|  | 23b | Discuss any limitations of the evidence included in the review. | Page 17 |
|  | 23c | Discuss any limitations of the review processes used. | Page 17 |
|  | 23d | Discuss implications of the results for practice, policy, and future research. | Page 14-17 |
| **OTHER INFORMATION** | | |  |
| Registration and protocol | 24a | Provide registration information for the review, including register name and registration number, or state that the review was not registered. | Page 6 |
|  | 24b | Indicate where the review protocol can be accessed, or state that a protocol was not prepared. | Page 6 |
|  | 24c | Describe and explain any amendments to information provided at registration or in the protocol. | Not applicable |
| Support | 25 | Describe sources of financial or non-financial support for the review, and the role of the funders or sponsors in the review. | Page 9 |
| Competing interests | 26 | Declare any competing interests of review authors. | Page 18 |
| Availability of data, code and other materials | 27 | Report which of the following are publicly available and where they can be found: template data collection forms; data extracted from included studies; data used for all analyses; analytic code; any other materials used in the review. | Page 18 |

*From:* Page MJ, McKenzie JE, Bossuyt PM, Boutron I, Hoffmann TC, Mulrow CD, et al. The PRISMA 2020 statement: an updated guideline for reporting systematic reviews. BMJ 2021;372:n71. doi: 10.1136/bmj.n71. This work is licensed under CC BY 4.0. To view a copy of this license, visit <https://creativecommons.org/licenses/by/4.0/>

## **Expanded Method S1. Full search strategy**

### **Ovid MEDLINE**

| 1 | exp artificial intelligence/ | 280419 |
| --- | --- | --- |
| 2 | exp natural language processing/ | 9282 |
| 3 | exp deep learning/ | 41475 |
| 4 | (artificial intelligence or computational intelligence or machine intelligence or machine learning or deep learning or hierarchical learning or transfer learning or reinforcement learning or computer vision or computer reasoning or automated reasoning or support vector machine* or support vector network* or neural network* or natural language processing or random forest* or decision tree* or knowledge representation* or perceptron*).tw,kw. | 490091 |
| 5 | 1 or 2 or 3 or 4 | 575117 |
| 6 | exp cardiovascular disease/ | 2939790 |
| 7 | exp cardiology/ | 25474 |
| 8 | exp cardiologist/ | 1489 |
| 9 | exp cerebrovascular accident/ | 200188 |
| 10 | (cardiovascular disease* or heart disease* or coronary artery disease* or angin* or ischaemic or ischemic or myocardial infarction* or STEMI or NSTEMI or acute coronary syndrome or atrial fibrillation* or ventricular fibrillation* or arrhythmia* or tachycard* or bradycard* or heart block or heart failure or systolic dysfunction or diastolic dysfunction or HFpEF or HFrEF or cardiomyopath* or valv* or aort* or mitral or cardiac arrest* or palpitat* or endocarditis or stenos?s or stroke or cerebrovascular accident or atheroscleros?s or arterioscleros?s or hypertens* or hyperlipid* or dyslipid* or cardiovascular risk* or coronary risk*).tw,kw. | 2750802 |
| 11 | (cardiolog* or echocardiog* or electrocardiog* or ECG or EKG or electrophysiolog* or interventional cardiolog* or structural cardiolog* or cardiac physiolog* or cardiac imag* or angiog* or cardiac computed tomograph* or cardiac magnetic resonance imag* or cardiac catheter* or stress test* or Holter monitor* or cardiac rehab*).tw,kw. | 988460 |
| 12 | 6 or 7 or 8 or 9 or 10 or 11 | 4415840 |
| 13 | exp controlled clinical trial/ | 752469 |
| 14 | exp randomization/ | 109688 |
| 15 | exp single blind procedure/ | 0 |
| 16 | exp double blind procedure/ | 0 |
| 17 | exp crossover procedure/ | 0 |
| 18 | (randomized controlled trial or randomised controlled trial or random allocation or allocated randomly or randomly allocated).tw,kw. | 224983 |
| 19 | 13 or 14 or 15 or 16 or 17 or 18 | 930231 |
| 20 | 5 and 12 and 19 | 1051 |
| 21 | exp Artificial Intelligence/ | 280419 |
| 22 | exp Natural Language Processing/ | 9282 |
| 23 | exp Deep Learning/ | 41475 |
| 24 | (artificial intelligence or computational intelligence or machine intelligence or machine learning or deep learning or hierarchical learning or transfer learning or reinforcement learning or computer vision or computer reasoning or automated reasoning or support vector machine* or support vector network* or neural network* or natural language processing or random forest* or decision tree* or knowledge representation* or perceptron*).tw,kf. | 494314 |
| 25 | 21 or 22 or 23 or 24 | 578718 |
| 26 | exp Cardiovascular Diseases/ | 2939790 |
| 27 | exp Cardiology/ | 25474 |
| 28 | exp Cardiologists/ | 1489 |
| 29 | exp Stroke/ | 200188 |
| 30 | (cardiovascular disease* or heart disease* or coronary artery disease* or angin* or ischaemic or ischemic or myocardial infarction* or STEMI or NSTEMI or atrial fibrillation* or ventricular fibrillation* or arrhythmia* or tachycard* or bradycard* or heart block or heart failure or systolic dysfunction or diastolic dysfunction or HFpEF or HFrEF or cardiomyopath* or valv* or aort* or mitral or cardiac arrest* or palpitat* or endocarditis or stenos?s or pacemaker or stroke or cerebrovascular accident or atheroscleros?s or arterioscleros?s or hypertens* or hyperlipid* or dyslipid* or cardiovascular risk* or coronary risk*).tw,kf. | 2790828 |
| 31 | (cardiolog* or echocardiog* or electrocardiog* or ECG or EKG or electrophysiolog* or interventional cardiolog* or structural cardiolog* or cardiac physiolog* or cardiac imag* or angiog* or "cardiac computed tomograph* or cardiac magnetic resonance imag*" or cardiac catheter* or stress test* or Holter monitor* or cardiac rehab*).tw,kf. | 993423 |
| 32 | 26 or 27 or 28 or 29 or 30 or 31 | 4443483 |
| 33 | exp Clinical Trial/ | 1047842 |
| 34 | exp Single-Blind Method/ | 36516 |
| 35 | exp Double-Blind Method/ | 189236 |
| 36 | exp Random Allocation/ | 109688 |
| 37 | exp Cross-Over Studies/ | 61576 |
| 38 | (randomized controlled trial or randomised controlled trial or random allocation or allocated randomly or randomly allocated).tw,kf. | 226014 |
| 39 | 33 or 34 or 35 or 36 or 37 or 38 | 1256628 |
| 40 | 25 and 32 and 39 | 1490 |

### **Ovid Embase**

| 1 | exp artificial intelligence/ | 201225 |
| --- | --- | --- |
| 2 | exp natural language processing/ | 20932 |
| 3 | exp deep learning/ | 114970 |
| 4 | (artificial intelligence or computational intelligence or machine intelligence or machine learning or deep learning or hierarchical learning or transfer learning or reinforcement learning or computer vision or computer reasoning or automated reasoning or support vector machine* or support vector network* or neural network* or natural language processing or random forest* or decision tree* or knowledge representation* or perceptron*).tw,kw. | 583313 |
| 5 | 1 or 2 or 3 or 4 | 661042 |
| 6 | exp cardiovascular disease/ | 6151742 |
| 7 | exp cardiology/ | 117951 |
| 8 | exp cardiologist/ | 33960 |
| 9 | exp cerebrovascular accident/ | 553771 |
| 10 | (cardiovascular disease* or heart disease* or coronary artery disease* or angin* or ischaemic or ischemic or myocardial infarction* or STEMI or NSTEMI or acute coronary syndrome or atrial fibrillation* or ventricular fibrillation* or arrhythmia* or tachycard* or bradycard* or heart block or heart failure or systolic dysfunction or diastolic dysfunction or HFpEF or HFrEF or cardiomyopath* or valv* or aort* or mitral or cardiac arrest* or palpitat* or endocarditis or stenos?s or stroke or cerebrovascular accident or atheroscleros?s or arterioscleros?s or hypertens* or hyperlipid* or dyslipid* or cardiovascular risk* or coronary risk*).tw,kw. | 4059903 |
| 11 | (cardiolog* or echocardiog* or electrocardiog* or ECG or EKG or electrophysiolog* or interventional cardiolog* or structural cardiolog* or cardiac physiolog* or cardiac imag* or angiog* or cardiac computed tomograph* or cardiac magnetic resonance imag* or cardiac catheter* or stress test* or Holter monitor* or cardiac rehab*).tw,kw. | 1521154 |
| 12 | 6 or 7 or 8 or 9 or 10 or 11 | 7341003 |
| 13 | exp controlled clinical trial/ | 1385642 |
| 14 | exp randomization/ | 102592 |
| 15 | exp single blind procedure/ | 97696 |
| 16 | exp double blind procedure/ | 328588 |
| 17 | exp crossover procedure/ | 111597 |
| 18 | (randomized controlled trial or randomised controlled trial or random allocation or allocated randomly or randomly allocated).tw,kw. | 345608 |
| 19 | 13 or 14 or 15 or 16 or 17 or 18 | 1619153 |
| 20 | 5 and 12 and 19 | 2575 |

### **Scopus**

Results: 2355

TITLE-ABS-KEY ( "artificial intelligence" OR "computational intelligence" OR "machine intelligence" OR "machine learning" OR "deep learning" OR "hierarchical learning" OR "transfer learning" OR "reinforcement learning" OR "computer vision" OR "computer reasoning" OR "automated reasoning" OR "support vector machine*" OR "support vector network*" OR "neural network*" OR "natural language processing" OR "random forest*" OR "decision tree*" OR "knowledge representation*" OR "perceptron*" ) AND TITLE-ABS-KEY ( "cardiovascular disease*" OR "heart disease*" OR "coronary artery disease*" OR angin* OR ischaemic OR ischemic OR "myocardial infarction*" OR STEMI OR NSTEMI OR "acute coronary syndrome" OR "atrial fibrillation*" OR "ventricular fibrillation*" OR arrhythmia* OR tachycard* OR bradycard* OR "heart block" OR "heart failure" OR "systolic dysfunction" OR "diastolic dysfunction" OR HFpEF OR HFrEF OR cardiomyopath* OR valv* OR aort* OR mitral OR "cardiac arrest*" OR palpitat* OR endocarditis OR stenos?s OR stroke OR "cerebrovascular accident" OR atheroscleros?s OR arterioscleros?s OR hypertens* OR hyperlipid* OR dyslipid* OR "cardiovascular risk*" OR "coronary risk*" OR cardiolog* OR echocardiog* OR electrocardiog* OR ECG OR EKG OR electrophysiolog* OR "interventional cardiolog*" OR "structural cardiolog*" OR "cardiac physiolog*" OR "cardiac imag*" OR angiog* OR "cardiac computed tomograph*" OR "cardiac magnetic resonance imag*" OR "cardiac catheter*" OR "stress test*" OR "Holter monitor*" OR "cardiac rehab*" ) AND TITLE-ABS-KEY ( "randomized controlled trial" OR "randomised controlled trial" OR "random allocation" OR "allocated randomly" OR "randomly allocated" OR "single blind*" OR "double blind*" OR crossover )

### **CENTRAL**

| ID | Search | Hits |
| --- | --- | --- |
| #1 | MeSH descriptor: [Artificial Intelligence] explode all trees | 4064 |
| #2 | (artificial intelligence or computational intelligence or machine intelligence or machine learning or deep learning or hierarchical learning or transfer learning or reinforcement learning or computer vision or computer reasoning or automated reasoning or support vector machine* or support vector network* or neural network* or natural language processing or random forest* or decision tree* or knowledge representation* or perceptron*):ti,ab,kw | 18335 |
| #3 | MeSH descriptor: [Cardiovascular Diseases] explode all trees | 162527 |
| #4 | ("cardiovascular disease" or "heart disease" or "coronary artery disease" or angin* or ischaemic or ischemic or "myocardial infarction" or STEMI or NSTEMI or "acute coronary syndrome" or "atrial fibrillation" or "ventricular fibrillation" or arrhythmia* or tachycard* or bradycard* or "heart block" or "heart failure" or "systolic dysfunction" or "diastolic dysfunction" or HFpEF or HFrEF or cardiomyopath* or valv* or aort* or mitral or "cardiac arrest" or palpitat* or endocarditis or stenos?s or stroke or "cerebrovascular accident" or atheroscleros?s or arterioscleros?s or hypertens* or hyperlipid* or dyslipid* or (cardiovascular NEXT risk*) or (coronary NEXT risk*)):ti,ab,kw | 340936 |
| #5 | MeSH descriptor: [Cardiology] explode all trees | 310 |
| #6 | (cardiolog* or echocardiog* or electrocardiog* or ECG or EKG or electrophysiolog* or (interventional NEXT cardiolog*) or (structural NEXT cardiolog*) or (cardiac NEXT physiolog*) or (cardiac NEXT imag*) or angiog* or (cardiac NEXT computed NEXT tomograph*) or (cardiac NEXT magnetic NEXT resonance NEXT imag*) or (cardiac NEXT catheter*) or (stress NEXT test*) or (Holter NEXT monitor*) or (cardiac NEXT rehab*)):ti,ab,kw | 103608 |
| #7 | #1 or #2 | 19745 |
| #8 | #3 or #4 or #5 or #6 | 413671 |
| #9 | #7 and #8 in Trials | 3353 |

### **Clinicaltrials.gov**

Search Results: 271 studies

Condition/Disease: "cardiovascular disease" OR "cardiovascular diseases" OR "heart disease" OR "heart diseases" OR "coronary artery disease" OR "coronary artery diseases" OR angina OR anginas OR ischaemic OR ischemic OR "myocardial infarction" OR "myocardial infarctions" OR STEMI OR NSTEMI OR “acute coronary syndrome” OR "atrial fibrillation" OR "atrial fibrillations" OR "ventricular fibrillation" OR "ventricular fibrillations" OR arrhythmia OR arrhythmias OR tachycardia OR tachycardias OR bradycardia OR bradycardias OR "heart block" OR "heart failure" OR "systolic dysfunction" OR "diastolic dysfunction" OR HFpEF OR HFrEF OR cardiomyopathy OR cardiomyopathies OR valve OR valves OR aorta OR aortic OR mitral OR "cardiac arrest" OR "cardiac arrests" OR palpitation OR palpitations OR endocarditis OR stenosis OR stenoses OR stroke OR "cerebrovascular accident" OR "cerebrovascular accidents" OR atherosclerosis OR arteriosclerosis OR hypertension OR hypertensive OR hyperlipidemia OR hyperlipidaemia OR dyslipidemia OR dyslipidaemia OR "cardiovascular risk" OR "coronary risk"

Intervention/Treatment: "artificial intelligence" OR "computational intelligence" OR "machine intelligence" OR "machine learning" OR "deep learning" OR "hierarchical learning" OR "transfer learning" OR "reinforcement learning" OR "computer vision" OR "computer reasoning" OR "automated reasoning" OR "support vector machine" OR "support vector machines" OR "support vector network" OR "support vector networks" OR "neural network" OR "neural networks" OR "natural language processing" OR "random forest" OR "random forests" OR "decision tree" OR "decision trees" OR "knowledge representation" OR "knowledge representations" OR "perceptron" OR "perceptrons"

No longer looking for participants: Active, not recruiting, Completed, Terminated studies

## **Expanded Method S2. Eligibility criteria for study selection**

Eligibility criteria developed following the PICOS framework**.**

| **PICOS+** | **Inclusion** | **Exclusion** |
| --- | --- | --- |
| **Population**  **(and Context)** | - Populations at risk of CVD or with established CVD. - Human clinicians or healthcare professionals (e.g., cardiac sonographer) involved in management of CVD and its risk factors. | - Studies on the use of AI for management of conditions other than CVD and its risk factors. - Studies involving non-human subjects (e.g., animal models) or preclinical simulations. |
| **Intervention** | - AI tools integrated into clinical decision-making or patient care, including screening, diagnosis, treatment, or prevention - We defined AI as computational methods using machine learning, deep learning, or data-driven algorithms that make predictions, classifications, or decisions, including but not limited to supervised and unsupervised machine learning, deep neural networks, natural language processing, and computer vision. | - Studies on rule-based systems or traditional statistical models. - Studies in which AI is not the main component of the intervention. - Studies in which AI is used solely for administrative, operational, documentation, or training purposes (e.g. speech-to-text transcription, appointment scheduling, data entry, medical education). - Studies on robot-assisted or virtual reality interventions. |
| **Comparator** | - The absence of AI tool, such as standard care or clinician without assistance of AI. | - Studies without a control group (i.e., single group studies) will be excluded. |
| **Outcomes** | - Outcomes related to patients (e.g., CVD events, cardiovascular risk factors, mortality, behavioural outcomes) or clinicians (e.g., procedural time, diagnostic accuracy, clinical workflow). | - Studies that do not report outcomes related to patients or clinicians. |
| **Study design** | - Randomized controlled trials (RCTs), prospective RCTs, cluster RCTs, and crossover RCTs will be included. | - Secondary analyses of RCTs, quasi-experimental studies (i.e., uncontrolled or controlled pre-post studies), pilot RCTs, feasibility studies, observational studies, mixed-methods studies with a quantitative component, qualitative studies, case series, animal studies, reviews, and expert opinion will be excluded. |
| **Other** | - Published in English. - Full-text article in a peer-reviewed journal or proceedings | - Published in a language other than English. - Abstracts, protocols, commentaries, editorials - Preprint articles that are not peer-reviewed (e.g., medRxiv, SSRN). |
| Abbreviations: PICOS+, population, intervention, comparator, outcome, study design, other; AI, artificial intelligence; RCTs, randomised controlled trials; CVD, cardiovascular disease; SSRN, Social Science Research Network. | | |

## **Table S3. List of excluded studies and reasons for exclusion**

| **Study** | **Reason for exclusion** |
| --- | --- |
| Dorsch, A., Thomas, S., Xu, C., Kaiser, W. and Dobkin, B., 2014. Implementation of a Multicenter, International, Randomized Clinical Trial in Subacute Stroke Patients Using Wireless Health Technology: Poster 41. Neurorehabilitation and Neural Repair, 28(4), p.N17. | Conference abstract |
| ClinicalTrials.gov [Internet]. Bethesda (MD): National Library of Medicine (US). 2000 Feb 29. Identifier NCT02599259, Using Artificial Intelligence on Mobile Devices to Automate Directly Observed Therapy (DOT), Confirm Medication Ingestion, and Optimize Treatment in Patients on Anticoagulation Therapy.; 2015 Nov 16. Available from: https://clinicaltrials.gov/ | Manually identified duplicate |
| Liu, X., 2018. A0069 Evaluating the impact of an integrated computer-based decision support with person-centered analytics for the management of hypertension: a randomized controlled trial. Journal of Hypertension, 36, p.e2. | Conference abstract |
| Ahmad, T., Desai, N.R., Yamamoto, Y., Biswas, A., Ghazi, L., Martin, M., Simonov, M., Dhar, R., Hsiao, A., Kashyap, N. and Allen, L., 2022. Alerting clinicians to 1-year mortality risk in patients hospitalized with heart failure: the REVEAL-HF randomized clinical trial. JAMA cardiology, 7(9), pp.905-912. | Wrong intervention |
| Park, A., Chute, C., Rajpurkar, P., Lou, J., Ball, R.L., Shpanskaya, K., Jabarkheel, R., Kim, L.H., McKenna, E., Tseng, J. and Ni, J., 2019. Deep learning–assisted diagnosis of cerebral aneurysms using the HeadXNet model. JAMA network open, 2(6), pp.e195600-e195600. | Wrong study design |
| Persell, S.D., Peprah, Y.A., Lipiszko, D., Lee, J.Y., Li, J., Ciolino, J.D., Karmali, K.N., DeLury, K. and Sato, H., 2019. Home Blood Pressure Monitoring Plus a Mobile Phone-Based Hypertension Health Coaching App or a Blood Pressure Tracking App: A Randomized Controlled Clinical Trial. Circulation, 140(Suppl_1), pp.A13403-A13403. | Manually identified duplicate |
| Toscos, T., Coupe, A., Wagner, S., Ahmed, R., Roebuck, A., Flanagan, M., Drouin, M. and Mirro, M., 2020. Engaging patients in atrial fibrillation management via digital health technology: the impact of tailored messaging. The Journal of Innovations in Cardiac Rhythm Management, 11(8), p.4209. | Wrong intervention |
| Wijnberge, M., Geerts, B.F., Hol, L., Lemmers, N., Mulder, M.P., Berge, P., Schenk, J., Terwindt, L.E., Hollmann, M.W., Vlaar, A.P. and Veelo, D.P., 2020. Effect of a machine learning–derived early warning system for intraoperative hypotension vs standard care on depth and duration of intraoperative hypotension during elective noncardiac surgery: the HYPE randomized clinical trial. Jama, 323(11), pp.1052-1060. | Wrong setting (disease/condition) |
| ClinicalTrials.gov [Internet]. Bethesda (MD): National Library of Medicine (US). 2000 Feb 29. Identifier NCT05117970, Artificial Intelligence-assisted Diagnosis and Prognostication in Low Ejection Fraction Using Electrocardiograms; 2021 Nov 11. Available from: https://clinicaltrials.gov/ | Manually identified duplicate |
| ClinicalTrials.gov [Internet]. Bethesda (MD): National Library of Medicine (US). 2000 Feb 29. Identifier NCT05118035, Applying an Artificial Intelligence-Enabled Electrocardiographic System for Reducing Mortality: a Pragmatic Randomized Clinical Trial; 2021 Nov 11. Available from: https://clinicaltrials.gov/ | Manually identified duplicate |
| Blomberg, S.N., Christensen, H.C., Lippert, F., Ersbøll, A.K., Torp-Petersen, C., Sayre, M.R., Kudenchuk, P.J. and Folke, F., 2021. Effect of machine learning on dispatcher recognition of out-of-hospital cardiac arrest during calls to emergency medical services: a randomized clinical trial. JAMA network open, 4(1), pp.e2032320-e2032320. | Wrong setting (disease/condition) |
| Gu S.-N.; Qin A.-H.; Zhao Y.; Zuo W.-Z.; Cao J.; Huang S.-Q. Follow-up of atrial fibrillation recurrence after ablation with a BigThumb electrocardiogram monitor. Academic Journal of Second Military Medical University, 2021;42(1):35EP - 40 | No full text available |
| Peabody, J.W., Paculdo, D., de Belen, E., Ganesan, D., Cooney, I. and Trujillo, N., 2023. Clinical utility of a novel test for assessing cardiovascular disease risk in type 2 diabetes: a randomized controlled trial. Diabetology & Metabolic Syndrome, 15(1), p.155. | Wrong intervention |
| Tian D.; Song L.; Sui X.; Wang J.; Du H.; Zhao R.; Wang Y.; Lu X.; Ma Z.; Xu Y.; Jin Z.; Song W. Clinical application value of deep learning reconstruction in low radiation dose CT pulmonary artery imaging. Chinese Journal of Radiology (China), 2022;56(5):563EP – 568. | No full text available |
| Burke, D.A., Corrigan, N., Herlihy, M., Nasaj, O., Dickson, J., Delaney, D. and Westrup, J., 2022. Real world evaluation of artificial intelligence echocardiography image guidance and acquisition with novice scanners in multiple clinical settings. European Heart Journal-Cardiovascular Imaging, 23(Supplement_1), pp.jeab289-011. | Wrong study design |
| Huang, X., Zhou, S., Ma, X., Jiang, S., Xu, Y., You, Y., Qu, J., Shang, H. and Lu, Y., 2023. Effectiveness of an artificial intelligence clinical assistant decision support system to improve the incidence of hospital-associated venous thromboembolism: a prospective, randomised controlled study. BMJ open quality, 12(4), p.e002267. | Wrong setting (disease/condition) |
| Kim, Y., Choi, A.D., Telluri, A., Lipkin, I., Bradley, A.J., Sidahmed, A., Jonas, R., Andreini, D., Bathina, R., Baggiano, A. and Cerci, R., 2023. Atherosclerosis Imaging Quantitative Computed Tomography (AI‐QCT) to guide referral to invasive coronary angiography in the randomized controlled CONSERVE trial. Clinical cardiology, 46(5), pp.477-483. | Wrong study design |
| Martinez-Gutierrez, J.C., Salazar-Marioni, S., Abdelkhaleq, R., Niktabe, A., Rodriguez-Quintero, J.C., Nahhas, M., Dhanjani, S., Ballekere, A., Iyyangar, A., Giancardo, L. and Dongarwar, D., 2023. Abstract TMP41: Machine Learning-Enabled Automated Large Vessel Occlusion Detection Improves Treatment Times: A Multi-Center Cluster Randomized Clinical Trial. Stroke, 54(Suppl_1), pp.ATMP41-ATMP41. | Conference abstract |
| Schuurmans J.; Rellum S.R.; Schenk J.; Van Der Ster B.J.P.; Van Der Ven W.H.; Geerts B.F.; Hollmann M.W.; Cherpanath T.G.V.; Lagrand W.K.; Wynandts P.; Paulus F.; Driessen A.H.G.; Terwindt L.E.; Eberl S.; Hermanns H.; Veelo D.P.; Vlaar A.P.J. 2023. HYPE-2-efficacy of the hypotension prediction index with diagnostic guidance on hypotension during elective cardiac surgery and postoperative intensive care unit admission-a randomised clinical trial. Intensive Care Medicine Experimental, 11(Supplement 1). | Conference abstract |
| Zhang, Y., Feng, Y., Sun, J., Zhang, L., Ding, Z., Wang, L., Zhao, K., Pan, Z., Li, Q., Guo, N. and Xie, X., 2024. Fully automated artificial intelligence-based coronary CT angiography image processing: efficiency, diagnostic capability, and risk stratification. European Radiology, 34(8), pp.4909-4919. | Wrong study design |
| Zhang, Q., Lin, Y., Zhang, H., Ding, J., Pan, J. and Zhang, S., 2024. The application value of a vendor-specific deep learning image reconstruction algorithm in “triple low” head and neck computed tomography angiography. Quantitative Imaging in Medicine and Surgery, 14(4), p.2955. | Wrong setting (disease/condition) |
| Kelshiker, M.A., Bachtiger, P., Mansell, J., Chhatwal, K., Almonte, M., Alrumayh, A., Zaman, J., Schmidt, A., Plymen, C.M., Kramer, D.B. and Peters, N.S., 2024. Seeing and believing: behavioural insights from implementing an artificial intelligence-enabled stethoscope in primary care. European Heart Journal, 45(Supplement_1), pp.ehae666-3489. | Conference abstract |
| Kumar, A., Baum, E., Parmer, C. and Kugler, J., 2025. Limited Echocardiogram Acquisition by Clinicians Aided with Deep Learning: A Randomized Controlled Trial. medRxiv, pp.2025-04. | Preprint manuscript |
| Keim-Malpass, J., Ratcliffe, S.J., Clark, M.T., Krahn, K.N., Monfredi, O.J., Hamil, S., Yousevfand, G., Jones, M.K., Nelson, A., Moorman, L.P. and Moorman, J.R., 2025. A pragmatic randomized controlled trial of artificial intelligence (AI)-based predictive analytics monitoring for early detection of clinical deterioration. medRxiv, pp.2025-01. | Preprint manuscript |
| Kasimovskaya, N., Krivetskaya, M., Geraskina, N., Ulianova, N., Chalova, E. and Shushpanova, A., 2025. Usefulness of Digital Smart Care in the Management of Systemic Hypertension. The American Journal of Cardiology, 250, pp.1-8. | Wrong intervention |
| Zhang, B., Wong, K.P., Liu, M., Hui, V., Guo, C., Liu, Z., Liu, Y., Xiao, Q. and Qin, J., 2025. Effect of artificial intelligence-based video-game system on dysphagia in patients with stroke: A randomized controlled trial. Clinical Nutrition, 45, pp.81-90. | Wrong setting (disease/condition) |
| Hu, B., Shi, Z., Lu, L., Miao, Z., Wang, H., Zhou, Z., Zhang, F., Wang, R., Luo, X., Xu, F. and Li, S., 2024. A deep-learning model for intracranial aneurysm detection on CT angiography images in China: a stepwise, multicentre, early-stage clinical validation study. The Lancet Digital Health, 6(4), pp.e261-e271. | Wrong setting (disease/condition) |
| Zhang, Y., Liu, Z., Cheng, Y., Wang, Z., Li, Z., Li, J. and Shuai, T., 2024. Deep learning image reconstruction for transcatheter aortic valve implantation planning: image quality, diagnostic performance, contrast volume and radiation dose assessment. Academic Radiology, 31(6), pp.2268-2280. | Wrong study design |
| Zisis, G., Carrington, M.J., Oldenburg, B., Whitmore, K., Lay, M., Huynh, Q., Neil, C., Ball, J. and Marwick, T.H., 2021. An m-Health intervention to improve education, self-management, and outcomes in patients admitted for acute decompensated heart failure: barriers to effective implementation. European Heart Journal-Digital Health, 2(4), pp.649-657. | Wrong intervention |
| Geng, Z., Chen, B., Li, Q., Han, X. and Zhu, X., 2022. Efficacy of morphine combined with mechanical ventilation in the treatment of heart failure with cardiac magnetic resonance imaging under artificial intelligence algorithms. Contrast Media & Molecular Imaging, 2022(1), p.1732915. | Wrong intervention |
| Reid, R.D., Morrin, L.I., Beaton, L.J., Papadakis, S., Kocourek, J., McDonnell, L., D'Angelo, M.E.S., Tulloch, H., Suskin, N., Unsworth, K. and Blanchard, C., 2012. Randomized trial of an internet-based computer-tailored expert system for physical activity in patients with heart disease. European journal of preventive cardiology, 19(6), pp.1357-1364. | Wrong intervention |
| Zamora, A., Carrion, C., Vazquez, G., Martin-Urda, A., Vilaseca, M., Paluzie, G., Sevilla, J.C., Soler, C., Plana, N. and Elosua, R., 2015, August. A clinical decision support system can improves the quality of lipid-lowering therapy in coronary patients. In European Heart Journal (Vol. 36, pp. 285-286). | Conference abstract |
| Segar, M.W., Patel, K.V., Keshvani, N., Kannan, V., Willett, D., Klonoff, D.C. and Pandey, A., 2025. Electronic Health Record Alert With Heart Failure Risk and Sodium Glucose Cotransporter 2 Inhibitor Prescriptions in Diabetes: A Randomized Clinical Trial. Journal of Diabetes Science and Technology, 19(6), pp.1496-1504. | Wrong intervention |
| Bian, P., Zhang, X., Liu, R., Li, H., Zhang, Q. and Dai, B., 2021. Deep‐Learning‐Based Color Doppler Ultrasound Image Feature in the Diagnosis of Elderly Patients with Chronic Heart Failure Complicated with Sarcopenia. Journal of Healthcare Engineering, 2021(1), p.2603842. | Wrong study design |
| Qiao, H.Y., Tang, C.X., Schoepf, U.J., Bayer 2nd, R.R., Tesche, C., Di Jiang, M., Yin, C.Q., Zhou, C.S., Zhou, F., Lu, M.J. and Jiang, J.W., 2022. One-year outcomes of CCTA alone versus machine learning–based FFRCT for coronary artery disease: a single-center, prospective study. European Radiology, 32(8), pp.5179-5188. | Wrong study design |
| ClinicalTrials.gov [Internet]. Bethesda (MD): National Library of Medicine (US). 2000 Feb 29. Identifier NCT05838456, Deep Learning Enabled Endovascular Stroke Therapy Screening in Community Hospitals; 2023 May 01. Available from: https://clinicaltrials.gov/ | Preliminary results only |
| ClinicalTrials.gov [Internet]. Bethesda (MD): National Library of Medicine (US). 2000 Feb 29. Identifier NCT05028179, PROTEUS: A PROspective Randomised Controlled Trial Evaluating the Use of Artificial Intelligence in Stress Echocardiology; 2021 Aug 31. Available from: https://clinicaltrials.gov/ | Manually identified duplicate |
| ClinicalTrials.gov [Internet]. Bethesda (MD): National Library of Medicine (US). 2000 Feb 29. Identifier NCT05117970, Artificial Intelligence-assisted Diagnosis and Prognostication in Low Ejection Fraction Using Electrocardiograms; 2021 Nov 11. Available from: https://clinicaltrials.gov/ | Manually identified duplicate |
| Current Controlled Trials [Internet]. London: BioMed Central. [date  unknown]. ISRCTN15113915, PROTEUS: Evaluating the use of artificial intelligence to support stress echocardiography testing for heart disease; 2021 July 09. Available from: https://doi.org/10.1186/ISRCTN15113915 | Manually identified duplicate |
| ClinicalTrials.gov [Internet]. Bethesda (MD): National Library of Medicine (US). 2000 Feb 29. Identifier NCT02599259, Using Artificial Intelligence on Mobile Devices to Automate Directly Observed Therapy (DOT), Confirm Medication Ingestion, and Optimize Treatment in Patients on Anticoagulation Therapy.; 2015 Nov 16. Available from: https://clinicaltrials.gov/ | Manually identified duplicate |
| Goldsmith, M.P., Nadkarni, V.M., Futterman, C., Gazit, A.Z., Baronov, D., Tomczak, A., Laussen, P.C. and Salvin, J.W., 2021. Use of a risk analytic algorithm to inform weaning from vasoactive medication in patients following pediatric cardiac surgery. Critical Care Explorations, 3(11), p.e0563. | Wrong study design |
| Zahid, M., Sughra, U., Mumtaz, S. and Hassan, M., 2023. Improving nutritional status of children using artificial intelligence-based mobile application postsurgery: randomised controlled trial. BMJ Nutrition, Prevention & Health, 6(2), p.367. | Wrong intervention |
| Adedinsewo DA, Morales-Lara AC, Dugan J, Garzon-Siatoya WT, Yao X, Johnson PW, Douglass EJ, Attia ZI, Phillips SD, Yamani MH, Tobah YB, Rose CH, Sharpe EE, Lopez-Jimenez F, Friedman PA, Noseworthy PA, Carter RE. Screening for peripartum cardiomyopathies using artificial intelligence in Nigeria (SPEC-AI Nigeria): Clinical trial rationale and design. Am Heart J. 2023 Jul;261:64-74. doi: 10.1016/j.ahj.2023.03.008. Epub 2023 Mar 25. PMID: 36966922. | Manually identified duplicate |
| ClinicalTrials.gov [Internet]. Bethesda (MD): National Library of Medicine (US). 2000 Feb 29. Identifier NCT05140642, Blinded Randomized Controlled Trial of Artificial Intelligence Guided Assessment of Cardiac Function; 2021 Dec 01. Available from: https://clinicaltrials.gov/ | Manually identified duplicate |
| Woodward G, Bajre M, Bhattacharyya S, Breen M, Chiocchia V, Dawes H, Dehbi HM, Descamps T, Frangou E, Fazakarley CA, Harris V, Hawkes W, Hewer O, Johnson CL, Krasner S, Laidlaw L, Lau J, Marwick T, Petersen SE, Piotrowska H, Ridgeway G, Ripley DP, Sanderson E, Savage N, Sarwar R, Tetlow L, Thompson B, Thulborn S, Williamson V, Woodward W, Upton R, Leeson P. PROTEUS Study: A Prospective Randomized Controlled Trial Evaluating the Use of Artificial Intelligence in Stress Echocardiography. Am Heart J. 2023 Sep;263:123-132. doi: 10.1016/j.ahj.2023.05.003. Epub 2023 May 14. PMID: 37192698. | Manually identified duplicate |
| ClinicalTrials.gov [Internet]. Bethesda (MD): National Library of Medicine (US). 2000 Feb 29. Identifier NCT04000087, Electrocardiogram Artificial Intelligence-Guided Screening for Low Ejection Fraction (EAGLE); 2019 June 27. Available from: https://clinicaltrials.gov/ | Manually identified duplicate |
| ClinicalTrials.gov [Internet]. Bethesda (MD): National Library of Medicine (US). 2000 Feb 29. Identifier NCT05118035, Applying an Artificial Intelligence-Enabled Electrocardiographic System for Reducing Mortality: a Pragmatic Randomized Clinical Trial; 2021 Nov 11. Available from: https://clinicaltrials.gov/ | Manually identified duplicate |
| Laranjo, L., T O’Hagan, E., Klimis, H., Marschner, S. and Chow, C.K., 2026. Effect of a mobile health intervention optimised with artificial intelligence on blood pressure and other cardiovascular risk factors in adults with high blood pressure: Rationale and design of My Intelligent Cardiac Assistant (MICArdiac) Randomised Controlled Trial. Open Heart, 13(1). | Trial protocol only |
| Berestova A, Klyueva Y, Gorozhanina E, Timoshina M. Hybrid Machine Learning Method and Standard Data Analysis Approaches for Predicting Treatment Outcomes of Cardiovascular Diseases: A Randomized Controlled Trial. La Clinica terapeutica. 2026;177(2):209-17. | Wrong intervention |
| Keim-Malpass J, Ratcliffe SJ, Clark MT, Krahn KN, Monfredi OJ, Hamil S, Yousefvand G, Jones MK, Nelson A, Moorman LP, Moorman JR. A randomized controlled trial of artificial intelligence-based analytics for clinical deterioration. Scientific Reports. 2026 Feb 5. | Wrong intervention |
| DeVore AD, Majmudar M, Etters L, Xie J, Hao C, Lam PH, Hernandez AF, Fonarow GC, Desai AS. Digital platform to optimize guideline-directed heart failure therapy: results of the AIM-POWER trial. Circulation: Heart Failure. 2026 Feb;19(2):e013231. | Wrong intervention |
| Mulder MP, Florax AA, Harmannij-Markusse M, Fresiello L, Donker DW, Potters JW. Comparison of a mean arterial pressure alarm to the hypotension prediction index in preventing intraoperative hypotension in elective moderate-to high-risk non-cardiac surgical patients: a study protocol for a blinded, parallel, randomized controlled trial with a non-inferiority framework. Trials. 2026 Jan 8. | Trial protocol only |
| Lin C, Lin CS, Chen SJ, Tsai SH, Sung CC, Chen CC, Hsu YJ, Hung YJ, Lin SH. AI-enabled electrocardiogram alert for potassium imbalance treatment: a pragmatic randomized controlled trial. Nature Communications. 2026 Jan 8;17(1):159. | Wrong setting (disease/condition) |
| Xu J, Yang Y, Zhao J, Li D, Zheng S, Gu J, Wang M. Innovative approaches for coronary heart disease management: integrating biomedical sensors, deep learning, and stellate ganglion modulation. Computer Methods in Biomechanics and Biomedical Engineering. 2026 Jan 2;29(1):85-102. | Wrong intervention |
| Yu X, Cao J, Xu J, Xu Q, Chen H, Yu D, Ou A, Hu Y, Ma L. Efficacy of Telemedical Interventional Management in Patients with Coronary Heart Disease Undergoing Percutaneous Coronary Intervention: Randomized Controlled Trial. Journal of medical Internet research. 2025 Oct 20;27:e63350. | Wrong intervention |
| Ba Z, Zhao S, Liu M, Chen G, Lian X, Yu F, Su Y, Wang Z, Yang L, Wang X, Zhang X. Effectiveness of an AI-enhanced management system for coronary heart disease (AIM-CHD): rationale and design of a single-centre, open-label, randomised, parallel-controlled trial. BMJ open. 2025 Sep 1;15(9):e105597. | Trial protocol only |
| Rakai E, Etminani F, Younan N, Andersson A, Andersson M, Vik T, Kunkel S, Sundin A, Holm J, Modica A, Linge HM. Systematic, randomized atrial fibrillation screening using detailed phenotyping with a risk prediction model combined with patch electrocardiogram in a Swedish population aged 65 years or older: the CONSIDERING-AF trial. Europace. 2025 Sep;27(9):euaf190. | Wrong intervention |
| Habibovic M, Douma E, Schäfer H, Sestayo-Fernandez M, Roovers T, Sun X, Schmidt H, Kotewitsch M, Widdershoven J, Cantarero-Prieto D, Mooren F. Patient-Centered risk prediction, prevention, and intervention platform (TIMELY) to support the continuum of Care in Coronary Artery Disease Using eHealth and artificial intelligence: protocol for a randomized controlled trial. JMIR Research Protocols. 2025 Aug 14;14(1):e66283. | Trial protocol only |
| Liu WT, Lin C, Lee CC, Chang CH, Fang WH, Tsai DJ, Lin WY, Hung Y, Chen KC, Lee CH, Tsai TN. Artificial Intelligence–Enabled ECGs for Atrial Fibrillation Identification and Enhanced Oral Anticoagulant Adoption: A Pragmatic Randomized Clinical Trial. Journal of the American Heart Association. 2025 Jul 15;14(14):e042106. | Manually identified duplicates |
| Budge J, Carrell T, Yaqub M, Wafa H, Waltham M, Pilecka I, Kelly J, Murphy C, Palmer S, Wang Y, Clough RE. The ARIA trial protocol: a randomised controlled trial to assess the clinical, technical, and cost-effectiveness of a cloud-based, ARtificially Intelligent image fusion system in comparison to standard treatment to guide endovascular Aortic aneurysm repair. Trials. 2024 Mar 25;25(1):214. | Trial protocol only |
| Rellum SR, Schuurmans J, Schenk J, van der Ster BJ, van der Ven WH, Geerts BF, Hollmann MW, Cherpanath TG, Lagrand WK, Wynandts P, Paulus F. Effect of the machine learning-derived hypotension prediction index (HPI) combined with diagnostic guidance versus standard care on depth and duration of intraoperative and postoperative hypotension in elective cardiac surgery patients: HYPE-2–study protocol of a randomised clinical trial. BMJ open. 2023 May 1;13(5):e061832. | Trial protocol only |
| Vedage NA, Friedman SF, Chang Y, Borowsky LH, Shah SJ, McManus DD, Atlas SJ, Singer DE, Lubitz SA, Maddah M, Ellinor PT. Risk-Guided Atrial Fibrillation Screening With Artificial Intelligence–Enabled Electrocardiogram Models: A VITAL-AF Trial Analysis. JACC. 2026 Apr 14;87(14):1798-813. | Wrong study design |
| Sakamoto A, Kagiyama N, Sato E, Nakamura Y, Murata A, Kaneko T, Miyazaki S, Ashikawa Y, Sugihara K, Minamino T. Artificial Intelligence–Based Automated Echocardiographic Analysis and the Workflow of Sonographers: A Randomized Crossover Trial (AI‐Echo RCT). Journal of the American Heart Association. 2026 Jan 6;15(1):e045637. | Manually identified duplicates |
| Gu Y, Xu W, Sun J, Zhang X, Zhu H. Application of pre-prescription review of suspicious data via intelligent supervision platform for secondary prevention of cardiovascular diseases based on evidence-based medicine. Advances in Interventional Cardiology/Postępy w Kardiologii Interwencyjnej. 2025;21(1). | Wrong intervention |
| Khurshid S, Friedman SF, Al-Alusi MA, Kany S, Sommers T, Anderson CD, Ho JE, McManus DD, Borowsky LH, Ashburner JM, Lubitz SA. Artificial intelligence-enabled analysis of handheld single-lead electrocardiograms to predict incident atrial fibrillation: an analysis of the VITAL-AF randomized trial. npj Digital Medicine. 2025 Nov 26. | Wrong study design |
| Joshi SR, Dharmalingam M, Vadavi A, Keshavamurthy A, Shamanna P, Shah L, Thajudeen M, Mohamed M. OR20-07 Digital Twin Technology For T2D Remission And ASCVD Risk Reduction: 2-Year RCT Outcomes. Journal of the Endocrine Society. 2025 Oct;9(Supplement_1):bvaf149-1032. | Conference abstract |
| Lilli A, Parollo M, Mazzocchetti L, De Sensi F, Rossi A, Notarstefano P, Santoro A, Aquaro GD, Cresti A, Lapira F, Faggioni L. Ventricular tachycardia ablation guided or aided by scar characterization with cardiac magnetic resonance: rationale and design of VOYAGE study. BMC Cardiovascular Disorders. 2022 Apr 14;22(1):169. | Trial protocol only |
| May AM, LoCoco S, Mikhova KM, Ghadban R, Cuculich PS, Cooper DH, Maddox TM, Thakkar P, Deych E, Rowlandson I, Siotis A. Design and Methods of the AUTOMATED-WCT Trial: Evaluating Machine Learning–Based ECG Support for WCT Interpretation. Current Problems in Cardiology. 2025 Sep 25:103186. | Trial protocol only |
| Lei L, Li J, Zhang L, Yuan X, Diao X, Qi L, Wang Y, Du W, Zhao W, Hu S. Design and rationale of the artificial intelligent dialogue System assisted comprehensive Management of secondary prevention Among post coronary aRtery bypass graft patienTs (SMART): protocol for a randomised controlled trial for postcoronary artery bypass grafting management. BMJ open. 2025 Nov 1;15(11):e106447. | Trial protocol only |
| Khandwalla RM, Shvartser A, Zimmer RJ, Chukumerije M, Share M, Zadikany R, Farkouh M, Fonarow G, Elad Y, Kittleson MM. Optimizing medical therapy using remote monitoring and artificial intelligence in patients with heart failure: the TURING-HF trial. Heart Failure. 2025 Aug 1;13(8):102504. | Wrong intervention |
| ClinicalTrials.gov [Internet]. Bethesda (MD): National Library of Medicine (US). 2000 Feb 29. Identifier NCT05893420, A Rapid Diagnostic of Risk in Hospitalized Patients Using Machine Learning; 2025 July 29. Available from: https://clinicaltrials.gov/ | Wrong study design |
| ClinicalTrials.gov [Internet]. Bethesda (MD): National Library of Medicine (US). 2000 Feb 29. Identifier NCT07008729, Heart Failure Patient Management and Interventions Using Continuous Patient Monitoring Outside Hospitals and Real-world Data (RETENTION); 2025 May 13. Available from: https://clinicaltrials.gov/ | Trial protocol only |

## **Figure S1. Temporal and geographical distribution of RCTs**

## **Table S4. Characteristics of AI interventions**

| **Study, Year** | **Intervention** | **Comparison** | **AI task** | **Data modality** | **AI Type** | **System Output** | **AI origins** | **Cited CONSORT-AI** |
| --- | --- | --- | --- | --- | --- | --- | --- | --- |
| Adedinsewo et al,^1^ 2024 | AI-digital stethoscope and AI-ECG algorithm | Standard 12-lead clinical ECG plus usual care | Screening for left ventricular systolic dysfunction | Single-lead ECG and phonocardiogram recordings (digital stethoscope) and 12-lead ECG waveform | CNN | Real-time binary predictions via mobile app | Academic (Mayo Clinic, USA) | Yes |
| Andre et al,^2^ 2023 | AI CT angiography assessment | Routine care | Detection and quantification of coronary lesion | Coronary CT angiogram images | Unspecified | Coronary segment labelling, lesion-specific images, and quantification results. | Industry (Siemens Healthcare, Germany) | No |
| Baum et al,^3^ 2023 | AI-guided POCUS device | Routine care | Guidance on apical-4-chamber image acquisition and interpretation | POCUS images | Unspecified | automatic Anatomical structures labels, real-time guidance on probe placement, and left ventricular systolic function estimation | Industry (Echonous) | No |
| Chen et al,^4^ 2021 | AI-enabled echocardiography | Routine echocardiography | Diagnosis of acute left heart failure in elderly patients | Echocardiography images | DCNN | Diagnostic classification (probability of disease state) | Academic (Zhejiang University) | No |
| De Backer et al,^5^ 2023 | AI-enabled CT computational modelling for planning of LAA closure | Routine care | AI–enabled anatomical analyses | Cardiac CT images | Unspecified | Simulations of different Amulet device sizes and implant positions | Industry (Feops, Belgium) | No |
| Deisenhofer et al,^6^ 2025 | AI-guided ablation of spatio-temporal dispersion areas plus PVI | Pulmonary vein isolation (PVI) only | Electrogram spatio-temporal dispersion detection | Multipolar intracardiac electrograms | XGBoost and CNN | Visual and audio cues representing areas of interest | Industry (Volta AF-Xplorer) | No |
| He et al,^7^ 2023 | AI assessment of transthoracic echocardiogram | Sonographer | Cardiac function assessment | Echocardiogram videos | CNN |  | Academic (EchoNet-Dynamic, Stanford) | Yes |
| Hill et al,^8^ 2022 | AI risk prediction algorithm plus diagnostic testing | Routine care | Atrial fibrillation risk prediction | Structured I data | Time-Varying neural network | AF risk score | Industry (Bristol-Myers Squibb) | No |
| Horne et al,^9^ 2022 | AI behaviour nudges | Routine care | Behavioural nudges for statin adherence | Structured data | Reinforcement learning | Computer-generated e-mails, text messages, and interactive voice response telephone call | Industry (CareCentra) | No |
| Huang et al,^10^ 2021 | AI-enabled handheld single-lead ECG monitor | Routine care | Detection of atrial fibrillation recurrences post-ablation | Single-lead ECG waveform | CNN and Bidirectional LSTM | Not stated | Industry (Shanghai Yueguang Medical Technologies, China) | No |
| Kelshiker et al,^11^ 2026 | Training and implementation in use of AI-stethoscopes in routine care | Routine care | Prediction of heart failure, atrial fibrillation, and valvular heart disease | Single-lead ECG and phonocardiogram waveforms | Unspecified | Real-time binary predictions (yes or no) corresponding to the three target conditions | Industry (Eko DUO, Eko Health Inc, CA, USA) | Yes |
| Kim et al,^12^ 2025 | AI quantitative coronary angiography–assisted PCI | Optical coherence tomography-guided PCI | Measurements of coronary artery stenosis severity and vessel dimensions | Coronary angiography images | CNN | Quantitative analytical data on lesion length, percent of diameter stenosis, minimal lumen diameter, proximal and distal diameter with average diameter value at reference segments | Industry (Medipixel, Inc) | No |
| Kumar et al,^13^ 2025 | POCUS device with integrated DL-functionality | Standard POCUS device | Real-time guidance for probe positioning and image quality assessment | B-mode ultrasound images | CNN | View classification, directional cues for probe positioning guidance, and real-time image quality assessment | Industry (Ultrasight^TM^) | No |
| Labovitz et al,^14^ 2017 | AI daily monitoring | Routine care | Stroke medication monitoring | Smartphone camera video data | Unspecified | Real-time dosing data and automated text messages or emails | Industry (AiCure, New York) | No |
| Lin et al,^15^ 2024a | AI-ECG assistance | Routine care | STEMI detection | 12-lead ECG waveform | CNN | SMS notifications and ECG images of potential STEMI cases | Academic (National Defense Medical Center, Taiwan) | No |
| Lin et al,^16^ 2024b | AI-ECG assistance | Routine care | Identification of hospitalised patients with a high risk of mortality | 12-lead ECG waveform | CNN | AI-ECG report with percentile score and warning messages to physicians | Academic (National Defense Medical Center, Taiwan) | Yes |
| Liu et al,^17^ 2025 | AI-ECG assistance | Routine care | Atrial fibrillation and atrial flutter detection | 12-lead ECG waveform | CNN | Brief alert message along with ECG image | Academic (National Defense Medical Centre, Taiwan) | Yes |
| Martinez-Gutierrez et al,^18^ 2023 | AI-enabled CT angiogram paired with mobile application | Routine care | Large vessel occlusion detection | CTA (computed tomography angiograph) images | CNN | Real-time alerts on mobile application | Industry (Viz) | No |
| O’Sullivan et al,^19^ 2026 | LLM-assisted clinical assessment (AMIE system) | Clinical assessment without LLM assistance | Clinical assessment | Clinical data summary | LLM | Clinical assessment results and management recommendations | Industry (Google Research, United States) | No |
| Persell et al,^20^ 2020 | Conversational artificial intelligence smartphone app plus home blood pressure monitor | Blood pressure tracking app plus home blood pressure monitor | Coaching on hypertension self-management | Unstructured data | Unspecified | Text notifications, in-app conversations | Industry (Lark Technologies, Inc) | No |
| Qian et al,^21^ 2025 | On-site deep learning-based CT-derived fractional flow reserve (CT-FFR) | Coronary CT angiography | Detection of lesion-specific ischaemia | CCTA images | CNN | CT-FFR values | Industry (Shukun Technology, China) | No |
| Sakamoto et al,^22^ 2026 | AI-assisted echocardiographic workflow | Standard workflow | Automated measurement of key echocardiographic parameters | Echocardiographic images | CNN | Automated measurements and trace lines overlaid on images | Industry (Us2.ai, Singapore) | No |
| Sato et al,^23^ 2025 | Sodium reduction program (genetic profile + AI-based app + educational information) | Control group (no intervention) and App-only group | Automated image processing of meals, estimation of salt intake, and provision of personalised advice based on user's input | Meals images | Unspecified | Analysis of nutritional contents | Industry (Wellmira Inc) | No |
| Trivedi et al,^24^ 2025 | Conversational AI phone calls | Routine care | Patient education and risk assessment | Patient voice and text responses | Unspecified | Dialogue flow and alerts | Industry (Health Management Systems, Inc) | No |
| Tsai et al,^25^ 2025 | AI-ECG assistance | Routine care | Screening for low ejection fraction | 12-lead ECG waveform | CNN | Continuous-valued predictions of EF | Academic (National Defense Medical Center, Taiwan) | Yes |
| Upton et al,^26^ 2024 | AI-augmented stress echocardiogram | Routine care | Screening for severe coronary artery disease | Stress echocardiogram images | CNN | Report with binary classification of severe coronary artery disease | Industry (Ultromics) | No |
| Wang et al,^27^ 2019 | AI screening of EHR | Routine care | Identification of anticoagulation underuse | Structured data | Not stated | Not stated | Academic (Harvard) | No |
| Yang et al,^28^ 2023 | On-site computed tomography–derived fractional flow reserve (CT-FFR) using machine learning | Routine care | Fractional flow reserve values simulation | Coronary Computed Tomography Angiography (CCTA) images | MLNN & BRNN | Continuous Fractional Flow Reserve (FFR) values (0–1) | Industry (DEEPVESSEL FFR, China) | No |
| Yao et al,^29^ 2021 | AI-ECG assistance | Routine care | Screening for low ejection fraction | 12-lead ECG waveform | CNN | AI-ECG Report with recommendation for echocardiogram | Academic (Mayo Clinic) | Yes |
| Yao et al,^30^ 2026 | AI-assisted remote rehabilitation plus routine health education | Routine verbal health education and conventional offline exercise guidance | Real-time motion capture and corrective feedback during exercise | Smartphone camera video frames | Unspecified | Real-time movement guidance and postexercise assessment reports | Industry (RaphAI Health Technologies Limited, China) | No |
| Zhang et al,^31^ 2026 | AI-based cerebrovascular disease clinical decision support system (CDSS) | Routine care | Automated segmentation of ischaemic lesions to provide infarct characteristics | Brain MRI images | DL | Infarct pattern and infarct characteristic parameters | Academic | Yes |
| Abbreviations: AI, artificial intelligence; CNN, convolutional neural network; CT, computed tomography; DL, deep learning; ECG, electrocardiogram; EF, ejection fraction; EHR, electronic health records; LLM, large language model; MRI, magnetic resonance imaging; PCI, percutaneous coronary intervention; STEMI, ST-elevation myocardial infarction. | | | | | | | |  |

## **Figure S2. Risk of bias of included trials**

Traffic light plot showing the risk of bias assessment of included trials across the five domains and their overall risk of bias.

## **Figure S3. Funnel plots**

Funnel plots for visualisation purpose only. Publication bias was not formally assessed using funnel plots asymmetry and Egger’s regression test because these methods are considered unreliable for meta-analyses of outcomes with a small number (<10) of studies. A= Heart failure diagnosis ; B= Echocardiogram utilisation; C= Systolic blood pressure ; D= Diastolic blood pressure; E= Body mass index ; F= Major adverse cardiovascular events; G = Atrial fibrillation diagnosis.

## **Expanded Method S3. Sensitivity analyses**

We conducted a series of sensitivity analyses to assess the robustness of the overall effect estimate. We used leave-one-out analyses to assess whether the direction of associations and statistical significance was influenced by a single large study with positive outcome. Leave-one-out analysis was not carried out for outcomes with two studies only. For outcomes including studies with high risk of bias, we reported the results of meta-analyses after excluding them. We also performed meta-analyses using fixed-effect model and compared the results with random-effects meta-analyses. For outcomes based on two or three studies only, we compared results of random-effects models estimated using the Wald-type and Hartung–Knapp–Sidik–Jonkman methods, as recommended in Cochrane Handbook for Systematic Reviews of Interventions.^32^

### **Major adverse cardiovascular events (MACE)**

In primary analysis, imaged-based AI-CDSS were associated with significant difference in major adverse cardiovascular events (RR 0.74 [95% CI 0.58–0.96]; p=0.04; I²=0%). Leave-one-out analysis indicated that the pooled estimate was sensitive to individual studies, as exclusion of any single study resulted in loss of statistical significance, with the calculated effect sizes (RR) ranging from 0.73 (95% CI 0.37–1.42; p=0.10) to 0.78 (95% CI 0.05–11.16; p=0.44). Sensitivity analysis using a fixed-effect model produced a similar pooled estimate with a narrower confidence interval (RR 0.74 [95% CI 0.65–0.85]; p<0.0001; I²=0%). Random-effects model with Wald-type method did not change the effect size of pooled estimate and statistical significance (RR 0.74 [95% CI 0.65–0.85]; p< 0.0001; I²=0%).

### **Systolic blood pressure**

In primary analysis, AI-based mHealth interventions were associated with significant reduction in systolic BP (mean difference -3.18 mm Hg [95% CI -5.50 to -0.86]; p=0.03; I²=0%). The direction of associations for systolic BP did not change with removal of any of the individual study, but the pooled effect estimates became statistically non-significant with removal of either Persell at el, 2020 (-3.50 mm Hg[95% CI -14.21 to 7.21]; p=0.15; I²=0%) or Sato et al, 2025 (-3.50 mm Hg [-14.21 to 7.21]; p=0.15; I²=0%). Sensitivity analysis using a fixed-effect model produced a similar pooled estimate with a narrower confidence interval and smaller p-value (mean difference -3.18 mm Hg [95% CI -5.27 to -1.10]; p=0.003; I²=0%). Random-effects model with Wald-type method did not change the effect size of pooled estimate but produced smaller p-value and narrower 95% confidence interval (mean difference -3.18 mm Hg [95% CI -5.27 to -1.10]; p=0.003; I²=0%).

### **Diastolic blood pressure**

In primary analysis, AI-based mHealth interventions were not associated with significant difference in diastolic BP (mean difference -1.30 mm Hg [95% CI -3.96 to 1.37]; p=0.17; I²=0%). Removal of Persell et al, 2020 changed the statistical significance and produced bigger effect size of the pooled estimates (mean difference -2.26 mm Hg [95% CI -3.12 to -1.39]; p=0.02; I²=0%). Sensitivity analyses using a fixed-effect model and random-effects model with Wald-type method produced pooled estimate and statistical significance that are similar to primary analysis (mean difference -1.30 mm Hg [95% CI -2.93 to 0.33]; p=0.12; I²=0%).

### **Body mass index**

In primary analysis, , AI-based mHealth interventions were not associated with significant difference in body mass index (mean difference -0.02 kg/m² [95% CI -0.52 to 0.57]; p=0.67; I²=0%). Sensitivity analysis using a fixed-effect model produced a similar pooled estimate with a narrower confidence interval (mean difference -0.02 kg/m² [95% CI -0.88 to 0.93]; p=0.96; I²=0%). Random-effects model with Wald-type method also did not change the effect size of pooled estimate and statistical significance (mean difference -0.02 kg/m² [95% CI -0.88 to 0.93]; p=0.96; I²=0%). Leave-one-out analysis was not performed as only two studies contributed to meta-analysis of this outcome.

### **Heart failure diagnosis**

In primary analysis, AI-enhanced ECGs did not result in significant difference in heart failure detection (RR 1.22 [95% CI 0.70–2.14]; p=0.34; I²=90%). The direction of associations and statistical significance did not change with removal of any of the individual study, with the calculated effect sizes ranging from 1.36 (RR [95% CI 1.00–1.85]) to 1.22 (RR [95% CI 0.40–3.73]). Statistical heterogeneity was reduced substantially (from I²=90% to I²=0%) after removal of Kelshiker et al, 2026, but not other individual studies. Removal of study with high risk of bias (Yao et al, 2021) did not change the direction of association and the statistical significance of pooled estimate (RR 1.22 [95% CI 0.40–3.73]; p=0.52; I²=89%). Sensitivity analysis using a fixed-effect model changed the direction of association and the statistical significance of pooled effect estimate (RR 0.89 [95% CI 0.84–0.95]; p=0.0003; I²=91%). Compared with primary analysis, random-effects model with Wald-type method showed identical pooled estimate and statistical significance (RR 1.22 [95% CI 0.87–1.71]; p=0.26; I²=90%).

### **Echocardiogram utilisation**

Primary analysis showed that AI interventions were not associated with increased echocardiogram utilisation (RR 1.03 [95% CI 0.69–1.53]; p=0.57; I²=46%). Effect size did not change when estimated using fixed-effect model (RR 1.03 [95% CI 0.99–1.08]; p=0.18; I²=46%) and random-effects model with Wald-type method (RR 1.03 [95% CI 0.96–1.09]; p=0.42; I²=46%), but confidence intervals were narrower. Summary effect remained statistically non-significant. Leave-one-out analysis was not performed as only two studies contributed to meta-analysis of this outcome.

### **Atrial fibrillation diagnosis**

The primary meta-analysis showed a non-significant increase in AF detection with AI (RR 1.27 [95% CI 0.49–3.28]; p=0.19; I²=0%). Both fixed-effect model and random-effects model with Wald-type method produced an identical pooled estimate, but with a narrower confidence interval and statistical significance (RR 1.27 [95% CI 1.06–1.53]; p=0.01; I²=0%). Leave-one-out analysis was not performed as only two studies contributed to meta-analysis of this outcome.

## **Table S5. Vote-counting of medication adherence**

| **Study** | **Follow-up duration**  **(months)** | **Measure of medication adherence** | **Threshold for adherence** | **Intervention versus control result** | **Between-group difference** |
| --- | --- | --- | --- | --- | --- |
| Labovitz et al, 2017 | 3 | Plasma drug concentration levels | Above the minimum required therapeutic range (C*_min_*) | 15/15 vs 6/12 | Favours intervention, p value not provided. |
| Persell et al, 2020 | 6 | No missed doses vs any missed doses using a 4-day recall | Full adherence | 87/108 vs 76/93 | Favours control, p>0.05 |
| Huang et al, 2021 | 12 | Unspecified | Unspecified | 25/49 vs 16/63 | Favours intervention, p<0.01 |
| Horne et al, 2022 | 12 | Proportion of days covered (PDC) | PDC ≥0.80 | 59/89 vs 47/93 | Favours intervention,  P<0.05 |
| Trivedi et al, 2025 | 6 | Days of missed doses in prescription medications over the past week | Zero doses of missed prescribed medications in past seven days | 54/82 vs 17/21 | Favours control, p>0.05 |

## **References**

1.	Adedinsewo DA, Morales-Lara AC, Afolabi BB, et al. Artificial intelligence guided screening for cardiomyopathies in an obstetric population: a pragmatic randomized clinical trial. *Nature Medicine* 2024; **30**(10): 2897–906.

2.	Andre F, Fortner P, Aurich M, et al. Human AI teaming for coronary CT angiography assessment: impact on imaging workflow and diagnostic accuracy. *Diagnostics* 2023; **13**(23): 3574.

3.	Baum E, Tandel MD, Ren C, et al. Acquisition of cardiac point-of-care ultrasound images with deep learning: a randomized trial for educational outcomes with novices. *CHEST Pulmonary* 2023; **1**(3): 100023.

4.	Chen J, Gao Y. The Role of Deep Learning‐Based Echocardiography in the Diagnosis and Evaluation of the Effects of Routine Anti‐Heart‐Failure Western Medicines in Elderly Patients with Acute Left Heart Failure. *Journal of Healthcare Engineering* 2021; **2021**(1): 4845792.

5.	De Backer O, Iriart X, Kefer J, et al. Impact of computational modeling on transcatheter left atrial appendage closure efficiency and outcomes. *Cardiovascular Interventions* 2023; **16**(6): 655–66.

6.	Deisenhofer I, Albenque J-P, Busch S, et al. Artificial intelligence for individualized treatment of persistent atrial fibrillation: a randomized controlled trial. *Nature Medicine* 2025: 1–8.

7.	He B, Kwan AC, Cho JH, et al. Blinded, randomized trial of sonographer versus AI cardiac function assessment. *Nature* 2023; **616**(7957): 520–4.

8.	Hill NR, Groves L, Dickerson C, et al. Identification of undiagnosed atrial fibrillation using a machine learning risk-prediction algorithm and diagnostic testing (PULsE-AI) in primary care: a multi-centre randomized controlled trial in England. *European Heart Journal-Digital Health* 2022; **3**(2): 195–204.

9.	Horne BD, Muhlestein JB, Lappé DL, et al. Behavioral nudges as patient decision support for medication adherence: the ENCOURAGE randomized controlled trial. *American Heart Journal* 2022; **244**: 125–34.

10.	Huang S, Zhao T, Liu C, et al. Portable device improves the detection of atrial fibrillation after ablation. *International Heart Journal* 2021; **62**(4): 786–91.

11.	Kelshiker MA, Bächtiger P, Petri CF, et al. Triple cardiovascular disease detection with an artificial intelligence-enabled stethoscope (TRICORDER) in the UK: a cluster-randomised controlled implementation trial. *The Lancet* 2026.

12.	Kim Y, Yoon H-J, Suh J, et al. Artificial Intelligence–Based Fully Automated Quantitative Coronary Angiography vs Optical Coherence Tomography–Guided PCI: The FLASH Trial. *Cardiovascular Interventions* 2025; **18**(2): 187–97.

13.	Kumar A, Baum E, Parmer-Chow C, Kugler J. Limited echocardiogram acquisition by novice clinicians aided with deep learning: A randomized controlled trial. *Biology Methods and Protocols* 2025; **10**(1): bpaf083.

14.	Labovitz DL, Shafner L, Reyes Gil M, Virmani D, Hanina A. Using artificial intelligence to reduce the risk of nonadherence in patients on anticoagulation therapy. *Stroke* 2017; **48**(5): 1416–9.

15.	Lin C, Liu W-T, Chang C-H, et al. Artificial intelligence–powered rapid identification of ST-elevation myocardial infarction via electrocardiogram (ARISE)—a pragmatic randomized controlled trial. *NEJM AI* 2024; **1**(7): AIoa2400190.

16.	Lin C-S, Liu W-T, Tsai D-J, et al. AI-enabled electrocardiography alert intervention and all-cause mortality: a pragmatic randomized clinical trial. *Nature Medicine* 2024; **30**(5): 1461–70.

17.	Liu WT, Lin C, Lee CC, et al. Artificial Intelligence–Enabled ECGs for Atrial Fibrillation Identification and Enhanced Oral Anticoagulant Adoption: A Pragmatic Randomized Clinical Trial. *Journal of the American Heart Association* 2025: e042106.

18.	Martinez-Gutierrez JC, Kim Y, Salazar-Marioni S, et al. Automated large vessel occlusion detection software and thrombectomy treatment times: a cluster randomized clinical trial. *JAMA neurology* 2023; **80**(11): 1182–90.

19.	O’Sullivan JW, Palepu A, Saab K, et al. A large language model for complex cardiology care. *Nature Medicine* 2026: 1–8.

20.	Persell SD, Peprah YA, Lipiszko D, et al. Effect of home blood pressure monitoring via a smartphone hypertension coaching application or tracking application on adults with uncontrolled hypertension: a randomized clinical trial. *JAMA network open* 2020; **3**(3): e200255–e.

21.	Qian Y, Chen M, Hu C, Wang X. CT-derived fractional flow reserve on therapeutic management and outcomes compared with coronary CT angiography in coronary artery disease. *British Journal of Radiology* 2025; **98**(1170): 956–64.

22.	Sakamoto A, Kagiyama N, Sato E, et al. Artificial Intelligence–Based Automated Echocardiographic Analysis and the Workflow of Sonographers: A Randomized Crossover Trial (AI‐Echo RCT). *Journal of the American Heart Association* 2026; **15**(1): e045637.

23.	Sato K, Inoue K, Yamaguchi T, et al. Sodium Reduction Program Incorporating Genetic Profile and an AI-Based App: A Randomized Clinical Trial. *JAMA network open* 2025; **8**(10): e2537540.

24.	Trivedi R, Laranjo L, Marschner S, et al. Conversational AI Phone Calls to Support Patients With Atrial Fibrillation: Randomized Controlled Trial. *JMIR cardio* 2025; **9**(1): e64326.

25.	Tsai D-J, Lin C, Liu W-T, et al. Artificial intelligence-assisted diagnosis and prognostication in low ejection fraction using electrocardiograms in inpatient department: a pragmatic randomized controlled trial. *BMC medicine* 2025; **23**(1): 342.

26.	Upton R, Akerman AP, Marwick TH, et al. PROTEUS: a prospective RCT evaluating use of AI in stress echocardiography. *NEJM AI* 2024; **1**(11): AIoa2400865.

27.	Wang SV, Rogers JR, Jin Y, et al. Stepped-wedge randomised trial to evaluate population health intervention designed to increase appropriate anticoagulation in patients with atrial fibrillation. *BMJ quality & safety* 2019; **28**(10): 835–42.

28.	Yang J, Shan D, Wang X, et al. On-site computed tomography–derived fractional flow reserve to guide management of patients with stable coronary artery disease: the TARGET randomized trial. *Circulation* 2023; **147**(18): 1369–81.

29.	Yao X, Rushlow DR, Inselman JW, et al. Artificial intelligence–enabled electrocardiograms for identification of patients with low ejection fraction: a pragmatic, randomized clinical trial. *Nature medicine* 2021; **27**(5): 815–9.

30.	Yao Q, Qiu B, He L, et al. Effects of Artificial Intelligence Recognition–Based Telerehabilitation on Exercise Capacity in Patients With Hypertension: Randomized Controlled Trial. *Journal of Medical Internet Research* 2026; **28**: e81400.

31.	Zhang X, Ding L, Jing J, et al. Effect of a clinical decision support system on stroke care quality and outcomes in patients with acute ischaemic stroke (GOLDEN BRIDGE II): cluster randomised clinical trial. *bmj* 2026; **392**.

32.	Deeks JJ, Higgins JPT, Altman DG, McKenzie JE, Veroniki AA (editors). Chapter 10: Chapter 10: Analysing data and undertaking meta-analyses [last updated November 2024]. In: Higgins JPT, Thomas J, Chandler J, Cumpston M, Li T, Page MJ, Welch VA (editors). Cochrane Handbook for Systematic Reviews of Interventions version 6.5. Cochrane, 2024. Available from cochrane.org/handbook.
